# Repeatability and Discriminatory Power of Chart-Based Visual Function Tests in Individuals With Age-Related Macular Degeneration: A MACUSTAR Study Report

**DOI:** 10.1001/jamaophthalmol.2022.2113

**Published:** 2022-06-23

**Authors:** Hannah M. P. Dunbar, Charlotte Behning, Amina Abdirahman, Bethany E. Higgins, Alison M. Binns, Jan H. Terheyden, Nadia Zakaria, Stephen Poor, Robert P. Finger, Sergio Leal, Frank G. Holz, Matthias Schmid, David P. Crabb, Gary S. Rubin, Ulrich F. O. Luhmann

**Affiliations:** 1 Department of Visual Neuroscience and Function, University College London Institute of Ophthalmology, London, United Kingdom; 2 Moorfields Eye Hospital National Health Service Foundation Trust, London, United Kingdom; 3 Medical Faculty, Institute of Medical Biometry, Informatics and Epidemiology, University of Bonn, Bonn, Germany; 4 Department of Optometry and Visual Sciences, School of Health Sciences, City, University London, London, United Kingdom; 5 Department of Ophthalmology, University of Bonn, Bonn, Germany; 6 Translational Medicine, Novartis Institute for Biomedical Research, Cambridge, Massachusetts; 7 Ophthalmology Research, Novartis Institute for Biomedical Research, Cambridge, Massachusetts; 8 Bayer AG, Berlin, Germany; 9 Roche Pharmaceutical Research and Early Development, Translational Medicine Ophthalmology, Roche Innovation Center Basel, Switzerland

## Abstract

**Importance:**

There is a need for validated clinical end points that are reliably able to quantify potential therapeutic effects of future treatments targeting age-related macular degeneration (AMD) before the onset of serious visual impairment.

**Objective:**

To assess the reliability and discriminatory power of 5 simple chart-based visual function (VF) tests as potential measures for clinical trial end points with regulatory and patient-access intention in intermediate AMD (iAMD).

**Design, Setting, and Participants:**

This international noninterventional study took place at 18 tertiary ophthalmology departments across Europe. Participants were recruited between April 2018 and March 2020 and were identified during routine clinical review. Participants with no AMD and early AMD were recruited from hospital staff, friends, and family of participants with AMD and via referrals from community ophthalmologists and optometrists. The repeatability and discriminatory power of 5 simple chart-based assessments of VF (best-corrected visual acuity [BCVA], low-luminance visual acuity [LLVA], Moorfields Acuity Test [MAT], Pelli-Robson Contrast Sensitivity [CS], and International Reading Speed Test [IReST]) were assessed in a repeated-measures design. VF assessments were performed on day 0 and day 14. Participants with early AMD, iAMD, late AMD, and no AMD were recruited.

**Main Outcomes and Measures:**

Intraclass correlation coefficients (ICCs) and Bland-Altman 95% limits of agreement (LoA) were computed to assess repeatability. Area under the receiver operating characteristic curves (AUCs) determined the discriminatory ability of all measures to classify individuals as having no AMD or iAMD and to differentiate iAMD from its neighboring disease states.

**Results:**

A total of 301 participants (mean [SD] age, 71 [7] years; 187 female participants [62.1%]) were included in the study. Thirty-four participants (11.3%) had early AMD, 168 (55.8%) had iAMD, 43 (14.3%) had late AMD, and 56 (18.6%) had no AMD. ICCs for all VF measures ranged between 0.88 and 0.96 when all participants were considered, indicating good to excellent repeatability. All measures displayed excellent discrimination between iAMD and late AMD (AUC, 0.92-0.99). Early AMD was indistinguishable from iAMD on all measures (AUC, 0.54-0.64). CS afforded the best discrimination between no AMD and iAMD (AUC, 0.77). Under the same conditions, BCVA, LLVA, and MAT were fair discriminators (AUC, 0.69-0.71), and IReST had poor discrimination (AUC, 0.57-0.61).

**Conclusions and Relevance:**

BCVA, LLVA, MAT, CS, and IReST had adequate repeatability in this multicenter, multiexaminer setting but limited power to discriminate between no AMD and iAMD. The prognostic power of these variables to predict conversion from iAMD to late AMD is being examined in the ongoing longitudinal part of the MACUSTAR study.

## Key Points

**Question:**

Under multicenter, multiexaminer conditions, do simple chart-based assessments of visual function (VF) have sufficient repeatability and discrimination in people with age-related macular degeneration (AMD) to be considered as measures for future clinical trial end points?

**Findings:**

In this cross-sectional study including 245 people with AMD and 56 healthy, age-similar control individuals, best-corrected visual acuity, low-luminance visual acuity, Moorfields Acuity Test, contrast sensitivity, and International Reading Speed Test had adequate repeatability but limited power to discriminate between no AMD and intermediate AMD (iAMD).

**Meaning:**

The findings suggest that the chart-based tests included in this study perform sufficiently well to be considered as potential measures for clinical trial end points; their prognostic power to predict conversion from iAMD to late AMD needs to be examined with longitudinal data.

## Teaser

This cross-sectional study evaluates the usefulness of common chart-based tests in terms of discrimination and repeatability as potential measures for end points in clinical trials.

## Introduction

Against the backdrop of an aging population, profound irreversible vision loss in atrophic age-related macular degeneration (AMD); frequent, costly, and invasive treatments for neovascular disease; and the economic, societal, and human burden of visual impairment caused by AMD, there is a serious unmet need for novel treatments that target AMD before the onset of significant visual impairment.^1,2^ Yet even if these treatments existed, relevant validated clinical end point measures that are reliably able to quantify potential therapeutic effects have not been established or accepted by regulators. Furthermore, the extent of visual decline in the earlier stages of AMD has not been fully identified, a necessary step in defining a treatment indication. These are both key elements in enabling clinical development of urgently needed therapies and making them available to patients.

The US Food and Drug Administration recommends change in visual function (VF) as a primary end point in trials assessing novel ocular therapeutics.^3^ Change in high-contrast best-corrected visual acuity (BCVA), specifically a change of 15 Early Treatment Diabetic Retinopathy Study (ETDRS) letters, has successfully been used as a primary end point in large, multicenter landmark trials in neovascular AMD over recent decades, leading to approval of antivascular endothelial growth factor treatments.^4,5^ However, BCVA has limited value as an end point to quantify early functional deficits in AMD when high-contrast visual acuity is good.^6^ Nevertheless, BCVA is the only validated visual function end point recognized by payers and regulators. This paucity of accepted and suitable end points has led to the initiation of several end point development trials, not just in AMD, but in inherited retinal dystrophies, where the ability to capture potential gene therapy response is crucial.^7,8,9,10,11^

Ideally, a clinical trial end point should be able to be measured simply and frequently, of low burden to patients and clinical sites, repeatable with negligible measurement error under real-word clinical conditions in large international multicenter trial settings, sensitive to longitudinal change and treatment effect, and clinically relevant and meaningful to patients.^6,11,12^ With 20 study sites, MACUSTAR presents an opportunity to consider how end points perform against these criteria.

MACUSTAR aspires to develop novel clinical trial end points within the areas of VF, structure, and patient-reported outcome with a regulatory and patient-access intention in intermediate AMD (iAMD). A full study design has been published.^8^ Briefly, structural and functional candidate end points are being evaluated in a cross-sectional cohort with respect to their repeatability and ability to distinguish normal aging changes from Beckman classified^13^ AMD severity stages. Subsequently, the capacity of candidate end points to track changes over time and to predict conversion from iAMD to late disease will be evaluated with 3-year longitudinal data.

The MACUSTAR consortium selected potential candidate end points in 2016 based on expert consensus and contemporaneous literature. End points demonstrating sufficient evidence to support their relevance in iAMD, data supporting adequate measurement quality, the expectation of successful repeated standardized administration across multiple sites, and acceptance by patients and examiners were included.^8,14^ This report focuses on a subset of the chosen VF end points that are chart based and arguably the simplest and least burdensome to capture, namely BCVA, low-luminance visual acuity (LLVA),^15^ Moorfields Acuity Test (MAT),^16^ Pelli-Robson Contrast Sensitivity (CS),^17^ and International Reading Speed Test (IReST).^18,19^ Under single-center, cross-sectional settings, all 5 chart-based tests have previously demonstrated a statistically significant reduction in visual function in participants with iAMD compared with age-similar healthy individuals.^20,21,22,23,24^ Whether this holds in large, multicenter investigations and whether they can track change over time remains to be seen. Here, using MACUSTAR cross-sectional data, we report on the repeatability and discriminatory power of these simple chart-based assessments of VF, and consider the feasibility of deploying these tests in future multicenter clinical trials.

## Methods

MACUSTAR recruited participants from 20 clinical centers. Participants from 18 European clinical sites contributed to the cross-sectional analysis presented here, as 2 sites began recruiting after the cross-sectional recruitment target had been met. From the 18 involved sites, 5 recruited participants with no AMD, early AMD, iAMD, and late AMD, whereas the remaining 13 sites recruited participants with iAMD only. Sample sizes were planned as follows: 50 with no AMD, 50 with early AMD, 50 with late AMD, and 150 with iAMD across 3 age categories (55-64 years, 65-74 years, and 75-85 years). The rationale for sample sizes has been described previously.^8^ Given the strong genetic background of AMD, differential genetic risk based on race, and to understand the generalizability of eventual longitudinal study results to other populations, race data were collected by self-report from the following categories: African, Asian, Caucasian, and other. Written informed consent was obtained from all participants. No incentives for participation were offered, but travel expenses were reimbursed. The research was approved by individual local ethics committees (summarized previously^25^) and conformed to the Declaration of Helsinki. Strengthening the Reporting of Observational Studies in Epidemiology (STROBE) reporting guidelines were followed.

Disease classifications were based on the Beckman classification system^13^ determined by a central reading center on the basis of multimodal imaging including color fundus photography, confocal infrared photography, fundus autofluorescence, and spectral-domain optical coherence tomography images obtained during a dedicated screening visit. Images were graded by a junior reader followed by a senior reader and reviewed according to a standardized predefined grading protocol.^26^ Inclusion and exclusion criteria have been previously published.^8,14^ Briefly, in addition to satisfying disease classification criteria, participants were required to be aged between 55 and 85 years and able to provide informed consent and comply with study visits. Those with concurrent ocular conditions in the study eye that in the opinion of the investigator would require surgical intervention to prevent or treat vision loss or that would affect interpretation of results were excluded, as were those with known systemic illnesses that would prevent participation and those with cognitive impairment or illiteracy or who did not speak the national language.

VF tests included BCVA, LLVA, MAT, CS, IReST (small-print standardized [SPS] and large-print standardized [LPS]), mesopic and scotopic microperimetry (S-MAIA; Centervue), and dark adaptation (AdaptDx; Maculogix). VF assessments were performed on day 0 (baseline) and again on day 14 (validation). This article reports on VF as measured by BCVA, LLVA, MAT, CS, and IReST, referred to herein as chart-based VF tests. The remaining device-based tests will be reported separately. A full description of examination procedures is provided in the eMethods in Supplement 1, including all standard operating procedures (SOP) and certification examination.

### Statistical Analysis

Repeatability was assessed by computing intraclass correlation coefficients (ICCs) with 95% CIs and Bland-Altman mean deviation (MD) with 95% limits of agreement (LoA).^27^ To investigate repeatability across sites, analyses were repeated on data from sites with at least 10 participants with iAMD and on a separate group of all participants with iAMD from remaining sites (eTable 1 in Supplement 1). Receiver operating characteristic (ROC) curves were used to examine the discriminatory ability of all baseline chart-based VF measures and low-luminance deficit^15^ to classify participants as having no AMD or iAMD and to differentiate iAMD from its neighboring disease states. All possible combinations of 2 chart-based VF tests were also considered. Area under the ROC curve (AUC) and 95% CIs (obtained from 2000 stratified bootstrap samples) were reported. Analyses were also performed for no AMD vs early AMD and for no AMD vs any AMD (early, intermediate, or late) (eTable 2 in Supplement 1). A full description of these statistical analyses are provided in the eMethods in Supplement 1.

## Results

A total of 301 participants (mean [SD] age, 71 [7] years; 187 [62.1%] female and 114 [37.9%] male; race data not reported to protect identity in low numbers) were recruited from 18 clinical sites (34 [11.3%] with early AMD, 168 [55.8%] with iAMD, 43 [14.3%] with late AMD, and 56 [18.6%] with no AMD). Baseline demographic characteristics and VF measures per disease group and for the full cohort are provided in Table 1. Deviations from planned sample sizes were the result of difficulty recruiting younger patients with late AMD and older patients with early AMD. The overall recruitment target was met by recruiting additional participants with no AMD and iAMD. Participants with no AMD were younger than those with iAMD (mean [SD] age, 68 [6] years vs 71 [8] years, respectively), and those with iAMD were younger than those with late AMD (mean [SD] age, 71 [8] years vs 75 [6] years, respectively). All participants had a refractive error of ±9.00 diopters (D) spherical equivalent.

**Table 1.** Summary of Demographic and Chart-Based Visual Function Measures at Baseline

Variable	AMD				Full cohort (n = 301)
	No. (n = 56)	Early (n = 34)	Intermediate (n = 168)	Late (n = 43)	
Age at baseline visit, y					
Mean (SD)	68 (6)	72 (6)	71 (8)	75 (6)	71 (7)
Median (range)	68 (55 to 88)	72 (57 to 82)	72 (55 to 88)	75 (64 to 84)	72 (55 to 88)
Gender, No. (%)					
Female	33 (58.9)	27 (79.4)	106 (63.1)	21 (48.8)	187 (62.1)
Male	23 (41.1)	7 (20.6)	62 (36.9)	22 (51.2)	114 (37.9)
**Best-corrected visual acuity, logMAR [Snellen]** ^a^					
Mean (SD)	−0.04 (0.08) [20/20]	0.01 (0.08) [20/20]	0.02 (0.10) [20/20]	0.77 (0.25) [20/125]	0.11 (0.30) [20/25]
Median (range)	−0.06 (−0.24 to 0.14) [20/16]	0.02 (−0.18 to 0.20) [20/20]	0.02 (−0.24 to 0.28) [20/20]	0.84 (0.20 to 1.24) [20/125]	0.02 (−0.24 to 1.24) [20/20]
**Low-luminance visual acuity, logMAR [Snellen]** ^a^					
Mean (SD)	0.14 (0.09) [20/25]	0.19 (0.14) [20/32]	0.24 (0.15) [20/32]	0.95 (0.24) [20/200]	0.31 (0.30) [20/40]
Median (range)	0.13 (−0.02 to 0.38) [20/25]	0.17 (−0.04 to 0.50) [20/32]	0.22 (−0.14 to 0.68) [20/32]	0.96 (0.52 to 1.52) [20/200]	0.22 (−0.14 to 1.52) [20/32]
**Low-luminance deficit, logMAR [Snellen]** ^a^					
Mean (SD)	0.18 (0.07) [20/32]	0.17 (0.10) [20/32]	0.21 (0.10) [20/32]	0.17 (0.25) [20/32]	0.20 (0.13) [20/32]
Median (range)	0.18 (0.02 to 0.32) [20/32]	0.17 (−0.02 to 0.42) [20/32]	0.20 (0.02 to 0.64) [20/32]	0.12 (−0.40 to 0.82) [20/25]	0.18 (−0.40 to 0.82) [20/32]
**Moorfields Acuity Test, logMAR [Snellen]** ^a^					
Mean (SD)	0.36 (0.11) [20/50]	0.42 (0.12) [20/50]	0.44 (0.14) [20/50]	1.03 (0.20) [20/200]	0.51 (0.26) [20/63]
Median (range)	0.35 (0.16 to 0.62) [20/50]	0.41 (0.20 to 0.72) [20/50]	0.42 (0.10 to 0.90) [20/50]	1.00 (0.66 to 1.48) [20/200]	0.42 (0.10 to 1.48) [20/50]
**Pelli-Robson contrast sensitivity, logCS **					
Mean (SD)	1.71 (0.16)	1.63 (0.16)	1.55 (0.17)	1.07 (0.34)	1.52 (0.28)
Median (range)	1.75 (1.05 to 1.95)	1.65 (1.25 to 1.90)	1.55 (1.05 to 1.95)	1.15 (0.20 to 1.55)	1.60 (0.20 to 1.95)
**Small-print IReST (words/min)**					
Mean (SD)	156 (38)	123 (44)	144 (41)	25 (36)	127 (58)
Median (range)	154 (77 to 293)	129 (51 to 215)	147 (31 to 285)	1 (0 to 132)	140 (0 to 293)
Missing, No. (%)^b^	1 (1.8)	0 (0)	15 (8.9)	4 (9.3)	20 (6.6)
**Large-print IReST (words/min)**					
Mean (SD)	168 (41)	134 (47)	151 (40)	32 (39)	136 (59)
Median (range)	168 (76 to 333)	138 (55 to 225)	156 (29 to 275)	11 (0 to 134)	149 (0 to 333)
Missing, No. (%)^b^	1 (1.8)	0 (0)	18 (10.7)	4 (9.3)	13 (4.3)

Abbreviations: AMD, age-related macular degeneration; IReST, International Reading Speed Test; logCS, logarithm of contrast sensitivity.

^a^ Snellen equivalents are approximate.

^b^ Twelve participants without access to language-appropriate IReST included in missing data rate.

Of the 301 participants, 290 attended both visits (28 with early AMD, 167 with iAMD, 41 with late AMD, and 54 with no AMD). Median (IQR) time between visits was 14 (12-18) days with no disease progression events observed during that period. All participants who attended both visits performed BCVA, LLVA, MAT, and CS twice. As no Danish version of the IReST is commercially available, 1 site did not perform this test. Of 289 participants with access to the IReST, 269 (93%) and 276 (96%) generated SPS and LPS measurements twice. The main reason for noncompletion was technician failure to request test performance (ie, forgotten or technician misunderstood). There were no cases of participant refusal.

**Figure 1.**
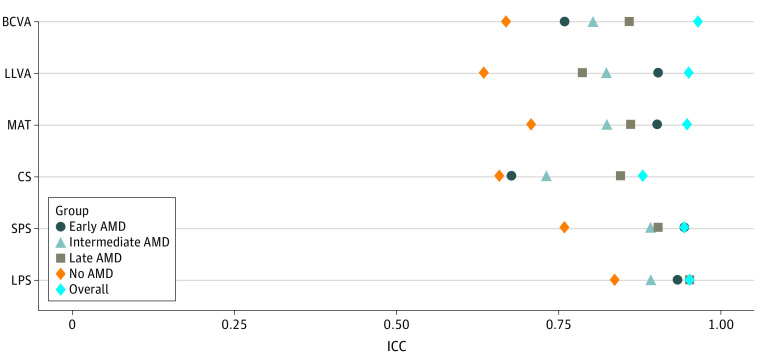
Intraclass Correlation Coefficients (ICCs) for the Full Cohort and Each Disease Group ICC values for the full cohort, no age-related macular degeneration (AMD), early AMD, intermediate AMD (iAMD), and late AMD groups. BCVA indicates best-corrected visual acuity; CS, contrast sensitivity; LLVA, low-luminance visual acuity; LPS, large-print standardized reading speed; MAT, Moorfields Acuity Test; SPS, small-print standardized reading speed.

Figure 1 plots ICC values for the full cohort and each individual disease group. ICCs for chart-based VF measures ranged between 0.88 (CS) and 0.96 (BCVA) when all data were considered, indicating good to excellent repeatability. ICCs calculated by disease group were slightly lower, with iAMD (0.73 [CS] to 0.89 [LPS and SPS]) and late AMD (0.79 [LLVA] to 0.95 [LPS]) groups exhibiting good reliability. Lowest ICCs were found in the no-AMD group (0.63 [LLVA] to 0.84 [LPS]). Bland-Altman plots were constructed for each chart-based VF test, considering data from all participants. Visual inspection revealed no evidence of heteroscedasticity, suggesting level of agreement is not associated with measurement scale. Table 2 provides all ICC and Bland-Altman metrics for the 4 disease groups.

**Table 2.** Intraclass Correlation (ICC) and Bland Altman (Mean Deviation and 95% Limits of Agreement [LoA]) Metrics for No Age-Related Macular Degeneration (AMD), Early AMD, Intermediate AMD (iAMD) and Late AMD Cohorts

Cohort	Chart-based visual function	(95% CI)			
		ICC	MD	Lower LoA	Upper LoA
No AMD	BCVA (logMAR)	0.67 (0.49 to 0.79)	0.01 (−0.01 to 0.01)	−0.12 (−0.15 to −0.09)	0.14 (0.11 to 0.17)
	LLVA (logMAR)	0.63 (0.44 to 0.77)	0.02 (0.00 to 0.04)	−0.15 (−0.19 to −0.11)	0.19 (0.15 to 0.23)
	MAT (logMAR)	0.71 (0.55 to 0.82)	0.02 (0.00 to 0.05)	−0.15 (−0.19 to −0.11)	0.20 (0.15 to 0.24)
	CS (logCS)	0.67 (0.48 to 0.79)	0.00 (−0.03 to 0.03)	−0.26 (−0.32 to −0.20)	0.27 (0.20 to 0.33)
	SP IReST (words/min)	0.76 (0.62 to 0.85)	−2 (−9 to 5)	−54 (−66 to −41)	49 (36 to 61)
	LP IReST (words/min)	0.84 (0.73 to 0.90)	5 (−1 to 11)	−39 (−49 to −28)	49 (38 to 59)
Early AMD	BCVA (logMAR)	0.76 (0.55 to 0.88)	0.00 (−0.02 to 0.02)	−0.10 (−0.14 to −0.06)	0.11 (0.07 to 0.14)
	LLVA (logMAR)	0.90 (0.80 to 0.95)	0.02 (0.00 to 0.04)	−0.10 (−0.14 to −0.06)	0.14 (0.10 to 0.18)
	MAT (logMAR)	0.90 (0.80 to 0.95)	0.01 (−0.02 to 0.03)	−0.10 (−0.13 to −0.06)	0.11 (0.07 to 0.14)
	CS (logCS)	0.68 (0.42 to 0.84)	−0.01 (−0.05 to 0.02)	−0.18 (−0.24 to −0.13)	0.16 (0.10 to 0.22)
	SP IReST (words/min)	0.94 (0.88 to 0.97)	0 (−7 to 6)	−33 (−44 to −22)	32 (21 to 43)
	LP IReST (words/min)	0.93 (0.86 to 0.97)	9 (3 to 15)	−22 (−33 to −12)	40 (30 to 51)
iAMD	BCVA (logMAR)	0.80 (0.74 to 0.85)	0.01 (0.00 to 0.02)	−0.11 (−0.13 to −0.10)	0.13 (0.12 to 0.15)
	LLVA (logMAR)	0.82 (0.77 to 0.87)	0.01 (0.00 to 0.02)	−0.16 (−0.18 to −0.14)	0.18 (0.16 to 0.20)
	MAT (logMAR)	0.82 (0.77 to 0.87)	0.02 (0.01 to 0.03)	−0.13 (−0.15 to −0.11)	0.17 (0.15 to 0.19)
	CS (logCS)	0.73 (0.65 to 0.79)	−0.01 (−0.03 to 0.01)	−0.27 (−0.30 to −0.23)	0.24 (0.20 to 0.27)
	SPS (words/min)	0.89 (0.85 to 0.92)	3 (0 to 7)	−34 (−39 to −29)	41 (35 to 46)
	LPS (words/min)	0.89 (0.85 to 0.92)	3 (0 to 6)	−33 (−38 to −28)	39 (34 to 44)
Late AMD	BCVA (logMAR)	0.86 (0.75 to 0.92)	0.04 (0.00 to 0.08)	−0.21 (−0.28 to −0.14)	0.30 (0.23 to 0.37)
	LLVA (logMAR)	0.79 (0.64 to 0.88)	0.03 (−0.01 to 0.07)	−0.27 (−0.36 to −0.19)	0.33 (0.25 to 0.42)
	MAT (logMAR)	0.86 (0.75 to 0.92)	0.01 (−0.02 to 0.05)	−0.20 (−0.26 to −0.14)	0.23 (0.17 to 0.29)
	CS (logCS)	0.85 (0.73 to 0.91)	−0.02 (−0.08 to 0.04)	−0.39 (−0.49 to −0.29)	0.35 (0.25 to 0.46)
	SP IReST (words/min)	0.90 (0.82 to 0.95)	3 (−2 to 9)	−27 (−26 to −18)	34 (25 to 43)
	LP IReST (words/min)	0.95 (0.91 to 0.98)	4 (1 to 8)	−17 (−23 to −11)	26 (20 to 32)

Abbreviations: BCVA, best-corrected visual acuity; CS, contrast sensitivity; LLVA, low-luminance visual acuity; logCS, logarithm of contrast sensitivity; LPS, large-print standardized; MAT, Moorfields Acuity Test; MD, mean deviation; SPS, small-print standardized.

No clinically relevant systematic bias or learning effects between visits were identified within any disease classification. MD for each disease group was within 2 letters for BCVA, LLVA, and MAT; less than 1 letter for CS; and 9 words per minute (wpm) or less for IReST measures. The LoA for letter scored tests were generally tighter within the no AMD, early AMD, and iAMD groups (±0.18 logMAR [9 letters] or less for BCVA, LLVA, and MAT and ±0.27 logCS [5.4 letters] or less for CS) compared with the late AMD cohort (±0.30 logMAR [15 letters] or less for BCVA, LLVA, and MAT and ±0.37 logCS [7.4 letters] or less for CS). For IReST measurements, the late AMD cohort demonstrated the tightest LoA of the disease groups (Table 2), though roughly 25% of participants with late AMD were unable to read any words at either sitting.

Given the multicenter nature of our data, ICC and Bland-Altman metrics were calculated for all study sites with 10 or more participants with iAMD and for a pooled group of 34 participants with iAMD at the remaining sites (eTable 1 in Supplement 1). Good to excellent ICC values were observed for 8 of 10 site groupings for BCVA, LLVA, and MAT; 7 of 10 site groupings for CS; 5 of 8 site groupings for SPS; and 7 of 8 site groupings for LPS. Corresponding LoA for each chart-based VF test were generally in line with those found for the iAMD cohort.

ROC curves were constructed to examine the discriminatory ability of each chart-based VF test. Analyses were performed with baseline data and again with validation data. As these resulted in equivalent findings, only baseline data are presented in Table 3. Discriminatory analysis results for no AMD vs early AMD and no AMD vs any AMD level (early, intermediate, or late) are provided in eTable 2 in Supplement 1.

**Table 3.** Receiver Operating Characteristic Analysis Summary for Early Age-Related Macular Degeneration (AMD) vs Intermediate AMD (iAMD), iAMD vs Late AMD, and No AMD vs iAMD

Chart-based VF	Early AMD vs iAMD			iAMD vs late AMD			No AMD vs iAMD		
	AUC^a^ (95% CI)	CV AUC		AUC (95% CI)	CV AUC		AUC (95% CI)	CV AUC	
		With VF	Without VF		With VF	Without VF		With VF	Without VF
BCVA	0.54 (0.43-0.64)	0.64	0.63	0.99 (0.99-1.00)	0.99	0.69	0.69 (0.61-0.76)	0.73	0.66
LLVA	0.60 (0.49-0.71)	0.67	0.63	0.99 (0.99-1.00)	0.99	0.69	0.71 (0.64-0.78)	0.74	0.66
LLD	0.60 (0.49-0.71)	0.66	0.63	0.63 (0.51-0.75)	0.70	0.69	0.59 (0.51-0.67)	0.68	0.66
MAT	0.56 (0.46-0.66)	0.65	0.63	0.99 (0.99-1.00)	0.99	0.69	0.70 (0.62-0.77)	0.72	0.66
CS	0.64 (0.54-0.73)	0.70	0.63	0.92 (0.88-0.96)	0.93	0.69	0.77 (0.70-0.84)	0.80	0.66
SPS	0.64 (0.54-0.75)	0.70	0.63	0.97 (0.95-1.00)	0.98	0.69	0.57 (0.48-0.66)	0.66	0.66
LPS	0.62 (0.51-0.74)	0.67	0.63	0.97 (0.95-0.99)	0.98	0.69	0.61 (0.52-0.70)	0.67	0.66

Abbreviations: AUC, area under the curve; BCVA, best-corrected visual acuity; CS, contrast sensitivity; CV, cross-validated; LLD, low-luminance deficit; LLVA, low-luminance visual acuity; LPS, large print standardized; MAT, Moorfields Acuity Test; SPS, small-print standardized; VF, visual function.

^a^ AUC values are provided for each chart-based visual function test for initial receiver operating characteristic and cross-validated receiver operating characteristic adjusted for age and sex, with and without the chart-based visual function measure included.

**Figure 2.**
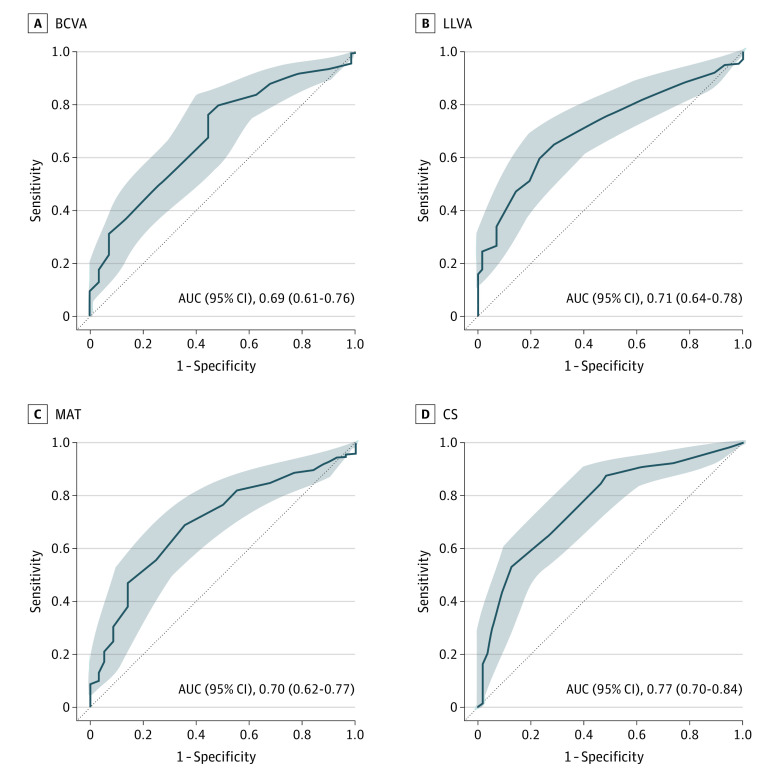
Receiver Operating Characteristic Curves Comparing No Age-Related Macular Degeneration (AMD) With Intermediate AMD for the 4 Best-Performing Chart-Based Visual Function Tests. Area under the curve (AUC) and 95% CIs (shading) for each curve are provided. BCVA indicates best-corrected visual acuity; CS, contrast sensitivity; LLVA, low-luminance visual acuity; MAT, Moorfields Acuity Test.

All measures displayed excellent discrimination between iAMD and late AMD (AUC, 0.92-0.99). By contrast, early AMD was indistinguishable from iAMD on all measures of chart-based VF (AUC, 0.54-0.64). CS afforded the best discrimination between no AMD and iAMD (AUC, 0.77). BCVA, LLVA, and MAT were fair discriminators under the same conditions (AUC, 0.69-0.71), whereas low-luminance deficit failed to discriminate between either group (AUC, 0.59). IReST reading speed measures also offered poor discrimination between no AMD and iAMD (AUC, 0.57-0.61). A combination of age and sex discriminated between disease groups as well as reading speed measures (Table 3). ROC curves for the 4 chart-based VF tests with best discrimination between no AMD and iAMD are provided in Figure 2.

AUC values for all possible combinations of 2 chart-based VF tests were determined, ranging from 0.53 to 0.75 for discrimination between no AMD and early AMD, 0.56 to 0.71 between early AMD and iAMD, and 0.58 to 0.79 between no AMD and iAMD. Of all combinations, the ability of CS and LLVA to discriminate between no AMD and iAMD was highest (AUC, 0.79, 95%CI: 0.73-0.86) but only marginally higher than CS alone.

## Discussion

To our knowledge, this is the first multicenter evaluation of the repeatability and discriminatory power of a battery of clinical chart-based VF tests, chosen for their promise as potential clinical end point measures for future iAMD treatment trials. Completion rates for all chart-based VF tests were high, reflecting their relative simplicity and demonstrating their feasibility within a multicenter iAMD cohort. Within the full cross-sectional MACUSTAR data set, ICC values for all chart-based VF tests ranged between 0.88 and 0.96, with only CS narrowly missing the 0.90 limit for excellent agreement. Largely good agreement was observed in the iAMD cohort, ranging between 0.73 and 0.89, in line with Chandramohan and colleagues^28^ who assessed a similar range of VF tests in a single center study with 20 participants. Also similar to Chandramohan et al,^28^ the lowest ICC values in our study were found in those with no AMD (Figure 1). This likely reflects the proportional influence of measurement range on ICCs,^29^ such that lower ICCs are expected in data sets with a smaller range.

iAMD LoA were largely equivalent to those defined in the no AMD group. Tightest limits were generally found in the early AMD group; however, differences when compared with the iAMD cohort were ±2.5 letters or less for tests scored by letter and less than ±5 wpm for IReST results and so not clinically meaningful. The widest LoA in letter-scored tests were observed in those with late AMD in keeping with previous reports in advanced eye disease,^30^ low vision,^31^ and late AMD.^32,33^ By contrast, IReST LoA in the late AMD group (SPS, ±31 wpm; LPS, ±22 wpm) were smaller than for any other disease stage; however, this was likely driven by approximately 25% of participants with late AMD achieving perfect agreement by failing to read any words at either visit.

There is complexity in drawing comparisons between repeatability studies owing to different metrics used (LoA and coefficient of repeatability) and the conditions under which data are collected. However, repeatability metrics derived from our multicenter, multitechnician data set compare favorably with previous reports, despite these reports predominantly being based on small, single-center, single-examiner studies. This likely reflects use of detailed SOPs, examiner certification, and ongoing data quality oversight in the current study.

LoA of approximately 1 line have been observed in healthy individuals for BCVA,^34,35,36^ LLVA,^37,38^ and MAT,^39^ comparable with the early AMD and iAMD cohorts in our study. Coefficients of repeatability of 9 letters (0.18 logMAR) and 8 letters (0.16 logMAR) have been shown for BCVA in individuals with early AMD and iAMD, respectively,^32^ compared with LoA of ±0.10 logMAR and ±0.12 logMAR in the corresponding cohorts in our study. Similarly, coefficients of repeatability of 0.13 logMAR^22^ and 12.21 letters (approximately 0.24 logMAR)^28^ have been reported for LLVA in iAMD, in line with LoA here of ±0.10 logMAR in early AMD and ±0.17 logMAR in iAMD. MAT LoA of approximately ±0.10 logMAR in participants with mixed AMD^16^ compare well with ±0.15 logMAR in our iAMD cohort. LoA for CS in normal observers are ±0.15 logCS (±3 letters),^40^ approximating our early AMD group (±0.17 logCS). Though our iAMD cohort were less repeatable at ±0.26 logCS, they mirrored coefficient of repeatability values of approximately 6 to 7 letters (0.28-0.36 logCS) reported previously in individuals with iAMD.^28,33,41^

Questionable repeatability of clinical reading tests has been noted as a concern for those planning clinical trials.^42^ In a study of VF tests in individuals with AMD,^41^ MNread reading speed^43^ was less repeatable than letter-based tests, such as BCVA and CS. Here, we obtained higher ICCs for IReST than BCVA, LLVA, and MAT within the iAMD group. We suggest the effect of a random reading mistake is likely less impactful across a paragraph-based test like IReST than a sentence-based test like MNread, which may account for these apparent differences in repeatability. ICCs and LoA observed here were roughly equivalent for small- and large-print versions of the IReST. To our knowledge, no previous MAT or IReST repeatability data in iAMD have been published.

As future phase 3 trials would almost certainly recruit participants from a large number of clinical centers, it is important to understand the performance of potential end point measures under such conditions. Because recruitment targets differed across sites, some sites did not have sufficient data to allow examination of site-specific repeatability (ie, fewer than 10 participants with iAMD). That said, adequate test-retest metrics across clinical sites, as demonstrated in eTable 1 in Supplement 1, suggest MACUSTAR chart-based VF SOPs allow collection of high-quality multicenter, multiexaminer data and supports their implementation in future iAMD trials.

All chart-based tests provide excellent discrimination between individuals with iAMD and those with late AMD (AUC, <0.92); however, their ability to discriminate between individuals with iAMD and healthy control individuals of a similar age is fair at best, with BCVA and IReST providing only poor discrimination. The ability of the same chart-based VF tests to discriminate between individuals with no AMD and those with iAMD was examined by Pondorfer et al^20^ in a single-center setting, with good levels of discrimination evident for CS, LLVA, and MAT). Though higher than AUC values presented here, both studies demonstrated greatest discrimination with CS, followed by LLVA, MAT, BCVA, and IReST in decreasing order. Narayanan et al^24^ also explored the discriminatory power of BCVA, LLVA, and CS between individuals with no AMD and those with nonadvanced AMD (AREDS grade, 1-4 on a simplified scale). Again, best discrimination was achieved with CS, with BCVA and LLVA failing to offer any discriminatory power. This may reflect the earlier disease staging of the nonadvanced AMD sample in their study.

Given that AMD stages are structurally defined, it is perhaps unsurprising that a single measure of VF offers no more than fair discrimination between no disease and iAMD. Furthermore, substantial functional heterogeneity has been previously noted within individuals in the early stages of AMD using low-luminance deficit, mesopic retinal sensitivity, and rod adaptation time.^22,23,44^ Additionally, international efforts to find a consensus definition for an OCT based classification are already under way.^45,46^ It will be critical to determine whether VF measures or combinations of VF and structural measures offer better discrimination between disease stages.

### Limitations

There are some limitations in this work. Test order was not randomized but performed in a specified order so that no 2 consecutive tests used the same letter series. Given the simplistic nature of these VF tests, minimal missing data, and lack of any learning effects, we are confident this structured order did not negatively impact our findings. Technicians were not masked to participants’ disease stage; however, as only 5 of 18 sites recruited across the disease severity groups, we do not anticipate a material impact on our findings. Phakic status was not considered as a possible confounder in our discriminatory analyses; however, having cataracts with the potential to considerably impact vision was a study exclusion criterion.^8^ Though disease groups were not age- or sex-matched, the overall sample remains representative of real-world disease presentation, with older people having more advanced disease and female individuals more likely to be affected than male individuals. We do not anticipate an impact on results, as repeatability analyses compares data within individuals, and discrimination analyses included age and sex as confounders.

VF test selection for MACUSTAR was limited by literature available in 2016. Since then, tablet-based tests of contrast and reading thresholds under mesopic conditions,^24^ and computer-based area under the log contrast sensitivity function determined using a quick algorithm under photopic and mesopic conditions have been associated with advancing stages of dry AMD.^47^ Furthermore, color-contrast tests reveal functional deterioration over a 12-month period in individuals with dry AMD.^44^ It remains to be seen how these tests fair under multiexaminer, multicenter settings.

## Conclusion

To our knowledge, we present the first evidence that BCVA, LLVA, MAT, CS, and IReST, simple chart-based measures of VF, have adequate repeatability in a multicenter, multiexaminer setting. We are cognizant that these favorable results likely reflect the use of SOPs, examiner certification, and ongoing data quality audits and would advocate this approach if these measures were to be adopted in future multicenter treatment trials. We hope publication of MACUSTAR SOPs and repeatability metrics prove a constructive contribution to those planning future trials within the iAMD space.

## Notes

Supplement 1. eMethods eTable 1. ICC and Bland Altman (mean deviation and 95% LoA) metrics for each clinical site with 10 or more iAMD participants and the pooled iAMD participants across remaining sites with fewer than 10 iAMD participants eTable 2. Receiver Operator Characteristic (ROC) analysis summary for no AMD versus early AMD and no AMD versus any AMD

Supplement 2. The MACUSTAR Consortium
