# Supplementary material for: Repeatability and Discriminatory Power of Chart-Based Visual Function Tests in Individuals With Age-Related Macular Degeneration: A MACUSTAR Study Report
Source: JAMA Ophthalmol. 2022 Jun 23;140(8):780–9. doi: 10.1001/jamaophthalmol.2022.2113 (PMC9227684; doi:10.1001/jamaophthalmol.2022.2113)
Supplement: Supplement 1. — eMethods eTable 1. ICC and Bland Altman (mean deviation and 95% LoA) metrics for each clinical site with 10 or more iAMD participants and the pooled iAMD participants across remaining sites with fewer than 10 iAMD participants eTable 2. Receiver Operator Characteristic (ROC) analysis summary for no AMD versus early AMD and no AMD versus any AMD [file jamaophthalmol-e222113-s001.pdf]

Supplemental Online Content

Dunbar HMP, Behning C, Abdirahman A, et al; the MACUSTAR Consortium. Repeatability and discriminatory power of chart-based visual function tests in individuals with age-related macular degeneration: a MACUSTAR study report. *JAMA Ophthalmol*. Published online June 23, 2022.
doi:10.1001/jamaophthalmol.2022.2113

eMethods

eTable 1. ICC and Bland Altman (mean deviation and 95% LoA) metrics for each clinical site with 10 or more iAMD participants and the pooled iAMD participants across remaining sites with fewer than 10 iAMD participants

eTable 2. Receiver Operator Characteristic (ROC) analysis summary for no AMD versus early AMD and no AMD versus any AMD

This supplemental material has been provided by the authors to give readers additional information about their work.

© 2022 Dunbar HMP et al. *JAMA Ophthalmology.*

eMethods

VISUAL FUNCTION EXAMINATION PROCEDURES AND STATISTICAL ANALYSES

Participants from 18 European clinical sites (6 German, 3 British, 2 Portuguese, 2 French, 2 Italian, 2 Dutch and 1 Danish) took part in this work. All chart-based visual function (VF) assessments were performed monocularly (right eye first) apart from IReST which was assessed for study eye only. Study eye was defined as that with better BCVA (at screening visit) if both eyes were eligible for enrolment. Where BCVA was equal in both eyes, study eye was selected by the investigator. Appropriate optical correction was provided as dictated by the test distance of each assessment.

Prior to the baseline and validation VF assessments, both eyes were refracted at 4 metres (m), reducing to 1m if insufficient letters were correctly identified at 4m (screening visit).

BCVA, LLVA and MAT utilise the Early Treatment of Diabetic Retinopathy (ETDRS) chart 1 and 2 letters series for right and left eye respectively. To reduce memory effects, the order of testing was standardised as follows: BCVA, CS, MAT, IReST and LLVA. In this way, consecutive tests used different letter series.

All testing was carried out by certified technicians in accordance with standard operating procedures (SOP). Certification involved review of all SOPs and successful completion of a 25-item multiple choice examination (pass mark 100%). Each site had a least two certified technicians. To mitigate any variation in test performance due to chart degradation and aging, new charts were provided to all sites, for all tests at the beginning of the study and appropriate storage and cleaning instructions provided.

Results of VF testing were recorded on custom case report forms (CRF) and transferred to an electronic CRF within 72 hours of the study visit. With the exception of refraction data, technicians were masked to previous examination results, though not to disease severity stage. Electronic CRF data was exported for data quality audit on a 6 monthly basis to identify potential training needs and to monitor missing data rates, timeliness of data entry, occurrence of outliers and potential data entry errors. Where such errors were suspected, a request for inputted data to be verified against source data was provided to the clinical site via the eCRF system. All requests were resolved before final data were exported in .tsv and .xlsx format to the study statistics team.

© 2022 Dunbar HMP et al. *JAMA Ophthalmology.*

Best-corrected visual acuity (BCVA), low luminance visual acuity (LLVA), Moorfields Acuity Test (MAT) were performed at 4m, reducing to 1m if insufficient letters were correctly identified from 4m. Participants were instructed to attempt all letters until at least 4 letters on one line were incorrectly read. Letter by letter scoring was employed. Charts were displayed in an ETDRS light box (Precision Vision, Woodstock, IL, USA) illuminated by 2 Cool Daylight 20-watt fluorescent tubes, burnt in for at least 96 hours prior to use. Testing was performed with room lights extinguished and windows covered. Chart luminance for BCVA and MAT was between 100 – 150 cd/m^2. To reduce chart luminance to the mesopic range, LLVA was performed with a mesopic filter (Precision Vision, Woodstock, IL, USA) inserted in front of the letter chart, reducing luminance to 3 cd/m^2, the standard used in the Food and Drug Administration refractive and intra-ocular lens outcome trials.[1, 2] Participants adapted to mesopic conditions for 10 minutes prior to testing. Care was taken to turn the lightbox off before removing either the chart or filter so as not to light adapt the participant.

Contrast sensitivity was performed at 1m using Pelli Robson (PR) charts 1 and 2 for right and left eyes respectively. The PR chart comprises 16 equal contrast letter triplets arranged in 8 rows of 2 triplets. Participants were instructed to attempt all letters until at least 2 letters in one triplet were incorrectly read. Letter by letter scoring was employed. Testing was performed with room lights on so that the luminance of the white portions of the chart was approximately 85 cd/m^2. Though it has been shown that differences in letter legibility across the PR chart can be minimised by accepting an "O" for a "C" and vice versa,[3] this was not employed here as to retain scoring method consistency with BCVA, LLVA and MAT examinations.

International Reading Speed Test (IReST) was performed in a habitual and standardised manner. Habitual measurements were performed binocularly with participant's habitual reading correction, at their habitual reading distance. Habitual reading speed data were collected to aid validation of a the Visual Impairment in Low Luminance questionnaire[4] and will be presented elsewhere. Standardised measurements were performed for the study eye only. Participants wore a trial frame containing their refraction result and a +2.50DS addition for a fixed 40cm working distance.

The IReST comprises 10 paragraphs of high contrast, proportionally spaced Times New Roman text containing approximately 150 words each. Each paragraph is centred on a page and displayed in a column formation imitating newsprint. Available in 18 languages, texts have been matched to ensure uniform word difficulty and syntactic complexity. IReST charts were available in 6 of the 7

© 2022 Dunbar HMP et al. *JAMA Ophthalmology.*

study languages (English, German, French, Portuguese, Dutch and Italian) at the time of study design. A Danish language version was not available so 12 Danish subjects did not perform the IReST.

The standard configuration of the IReST uses 9-point sized print. To ensure adequate data collection from participants with late AMD, a second 18-point version of the chart was custom made by Precision Vision for the MACUSTAR study. All other design concepts were uniform across the 9 point and 18 point versions. Following a practice run, participants were instructed to read aloud 1 randomly selected paragraph of each size from a 40cm viewing distance with appropriate optical correction. Different texts were used for each visit. Test illumination was between 300 – 400 lux. IReST texts were covered from the participant's view during test set up and explanation. The cover was removed at the start of the test and a stopwatch started. The time taken to read the paragraph and any errors made were recorded. Standardised reading speed in words per minute was calculated based on the number of correctly read words. This resulted in two reading speed measures, small print standardised (SPS) and large print standardised (LPS). The time taken to perform a refraction plus all chart-based tests was reported by site technicians to be approximately 40-45 minutes, with participants requiring a 1m test distance taking longer due to the need to adjust the test distance during examinations.

BCVA, LLVA, and MAT letter scores were converted to corresponding LogMAR values as follows:

LogMAR value = 1.7 – (letter score x 0.02)

CS letter score was converted to its corresponding LogCS value as:

LogCS value = (letter score – 3) x 0.05

Prior to analysis, one additional variable, Low Luminance Deficit (LLD)[5] was calculated as:

LLD = BCVA (logMAR) - LLVA (logMAR)

Therefore, the chart-based VF metrics under analysis were BCVA, LLVA, LLD, and MAT on a LogMAR scale, CS on a logCS scale, and IReST small print standardised (SPS) and large print standardised (LPS) reading speeds on a words per minute (wpm) scale.

Normal quartile plots were constructed for all chart-based VF measures. As no gross departures from a normal distribution were observed, parametric statistical methods were used. Repeatability

© 2022 Dunbar HMP et al. *JAMA Ophthalmology.*

was evaluated for all directly measured metrics (BCVA, LLVA, MAT, CS, SPS and LPS) by comparing data obtained during validation visit to those obtained at baseline (baseline – validation). Complete case analysis was performed. Using R package 'irr' (Matthias Gamer, Jim Lemon and Ian Fellows Puspendra Singh [2019]. irr: Various Coefficients of Interrater. Reliability and Agreement. R package version 0.84.1. https://CRAN.R-project.org/package=irr), intra-class correlation coefficients (ICC) with 95% confidence intervals (CI) were computed. For calculation of ICCs a one-way model ("ICC(1)") that regards only participants as random effects (as not every technician assessed each participant) was used. ICCs were calculated for all subjects and individual disease severity groups. Results were interpreted against the following guidelines; ICCs < 0.50 indicated poor reliability, ICCs between 0.50 and 0.75 indicated moderate reliability, ICCs between 0.75 and 0.90 indicated good reliability and ICCs between >0.90 indicated excellent reliability.[6] Bland-Altman plots were generated for the same cohorts. Mean deviation (MD) defined as baseline - validation visit was computed to assess bias. Additionally 95% limits of agreement (LoA) were calculated as $\pm$ 1.96 times the standard deviation (SD) of between visit differences.[7] It is expected that 95% of the time, the difference between 2 measurements will fall within the 95% LoA.[8] To investigate repeatability across sites, these analyses were repeated on data from sites with at least 10 iAMD participants and on a separate group of all iAMD participants from remaining sites. A by-technician analyses was not possible as technician identifiers were not captured by the eCRF.

Receiver operating characteristic (ROC) curves were used to examine the discriminatory ability of all chart-based VF measures obtained at baseline, plus low luminance deficit (LLD)[5] to classify subjects as having no AMD or i-AMD and to differentiate i-AMD from its neighbouring disease states (early AMD from i-AMD and i-AMD from late AMD). All possible combinations of two chart-based VF tests were also considered. Area under the ROC curve (AUC) with 95% CIs (obtained from 2000 stratified bootstrap samples) were reported. AUC values support comparison of the discriminatory power between tests (or combination of two tests) where 1 equates to perfect discrimination between groups.[9] For combinations of VF tests, multiple logistic regression models were built using disease state as the outcome variable and 2 VF measures as independent variables. Five-fold cross-validated ROC curves and AUC values were computed to evaluate the discriminatory ability of all models. Values greater than 0.9 indicated excellent discrimination, 0.8 – 0.9 good, 0.7 – 0.8 fair, 0.6 – 0.7 poor and 0.6 or less represented a failure to discriminate.[9] To further explore the discriminatory ability of each chart-based VF measure, two logistic regression models were fitted with disease severity stage as an outcome variable and sex and age as possible confounders. The first contained the respective chart-based VF measure as a covariate, while the second did not. In

this way, the difference between the two resultant AUC values indicates the influence of each specific chart-based VF test on discriminatory power over and above that of age and sex. Model fitting and evaluation was performed as described above.

1. Evans, D.W. *FDA update: contrast sensitivity testing standards. Ophthamol Manage February 2005. Available at: http://www.ophthalmologymanagement.com/articleviewer.aspx?articleid=86281.* 2005 10th May 2021].
2. Institute, A.N.S., *American National Standard for Phakic Intraocular Lenses. ANSI Z80.13-2007 (R2012)*. 2007, American National Standards Institute: Arlington, VA.
3. Elliott, D.B., D. Whitaker, and L. Bonette, *Differences in the legibility of letters at contrast threshold using the Pelli-Robson chart.* Ophthalmic and Physiological Optics, 1990. **10**(4): p. 323-326.
4. Pondorfer, S.G., et al., *Development of the Vision Impairment in Low Luminance Questionnaire.* Translational Vision Science & Technology, 2021. **10**(1): p. 5-5.
5. Sunness, J.S., et al., *Low luminance visual dysfunction as a predictor of subsequent visual acuity loss from geographic atrophy in age-related macular degeneration.* Ophthalmology, 2008. **115**(9): p. 1480-1488. e2.
6. Koo, T.K. and M.Y. Li, *A guideline of selecting and reporting intraclass correlation coefficients for reliability research.* Journal of chiropractic medicine, 2016. **15**(2): p. 155-163.
7. Bland, J.M. and D. Altman, *Statistical methods for assessing agreement between two methods of clinical measurement.* The lancet, 1986. **327**(8476): p. 307-310.
8. Myles, P.S. and J. Cui, *I. Using the Bland–Altman method to measure agreement with repeated measures*. 2007, Oxford University Press.
9. Pines, J.M., et al., *Evidence-based emergency care: diagnostic testing and clinical decision rules*. Vol. 83. 2013: John Wiley & Sons.

Refraction

Standard Operating Procedure

MACUSTAR Study
ECR-AMD-2017-13

© 2022 Dunbar HMP et al. *JAMA Ophthalmology.*

Distribution

This SOP is part of the Manual of Study Procedures for the Clinical Study MACUSTAR and its distribution is performed by AIBILI in a controlled manner.

1. PURPOSE

This document describes the procedures to perform Refraction for the Clinical Study *"Development of novel clinical endpoints for interventional clinical trials with a regulatory and patient access intention in patients with intermediate age-related macular degeneration*

© 2022 Dunbar HMP et al. *JAMA Ophthalmology.*

(AMD) – MACUSTAR”(Protocol nº ECR-AMD-2017-13) to ensure that a uniform procedure is followed by all clinical sites (CS) participating in the study, in order to obtain comparable and reliable data, as according to International Conference on Harmonization Good Clinical Practice (ICH-GCP). This procedure will be performed to the study subjects according to the Clinical Study Protocol.

2. POLICY/SCOPE

This SOP will be used when performing a refraction, to ensure all procedures are performed consistently. Adherence to this SOP is necessary to ensure accuracy of refraction results, which in turn influence the quality of visual function measures and subsequent data analysis. Adherence to this SOP will also ensure proper treatment of subjects, and that all data are available for future analysis.

3. STAFF TRAINING AND RESPONSIBILITIES

The Principal Investigator is responsible for ensuring that the appropriate personnel for performing refraction is identified and trained. All technicians should read this procedure before starting the participation in the Study. Clinical Sites are recommended to have a minimum of 2 technicians in the investigational team for this procedure. Technicians will be certified as follows:

a) Each technician must read this SOP and the SOPs related to Best Corrected Visual Acuity, Moorfields Acuity Test, Low Luminance Acuity, Contrast Sensitivity and Standardised IReST assessments.
b) Each technician will complete the Visual Function Testing Certification Examination prior to undertaking any subject testing. This multiple choice question exam covers the certification process for Refraction, Best Corrected Visual Acuity, Moorfields Acuity Test, Low Luminance Acuity, Contrast Sensitivity and Standardised IReST assessments.
c) A pass mark of 100% is required to be certified to undertake Best Corrected Visual Acuity, Moorfields Acuity Test, Low Luminance Acuity, Contrast Sensitivity and Standardised IReST assessments. The exam can be repeated until a 100% grade is achieved.

© 2022 Dunbar HMP et al. *JAMA Ophthalmology.*

4. PROCEDURE

4.1 Equipment

This procedure requires use of the following equipment:

Early Treatment of Diabetic Retinopathy Study (ETDRS) fluorescent tube illuminated light box with 5-pronged wheel base
ETDRS letter chart R
Subject chair with backrest in fixed position
Refraction Score Sheet
Pen
Lux meter
Trial frame
Open aperture trial lens set
Autorefractor or retinoscope
Jackson cross cylinders $\pm$0.25, $\pm$0.50, $\pm$1.00
Cotton pads
Surgical tape
Focimeter or lensmeter
Alcohol hand rub
Alcohol wipes

4.1.1 Equipment Maintenance

The supplier recommendation for the equipment should be followed for maintenance. Only qualified and trained personnel should do service and repair. All interventions in the equipment should be recorded, kept and made available during monitoring visits or audits if requested.

4.2 Subject Information

Oral information regarding the examination that is being to be performed is given to the subject.

4.3 Technical Procedure

Refraction is a method used to determine the best spectacle correction for a patient to perform visual tasks at specified distances. It involves and subjective techniques to determine the most positive or least negative spherical and least negative cylindrical lens consistent with best corrected visual acuity.

© 2022 Dunbar HMP et al. *JAMA Ophthalmology.*

4.3.1 Considerations before starting tests

a) Subject identification

All data must be recorded on the Refraction Score Sheet. The subject's study identification code, study eye, date of examination and study visit should be recorded where requested.

b) Pupil size

There are no specific pupil size requirements, but pupils must not be dilated.

c) Testing room dimensions

Refraction is carried out in a room that allows a 4 meter test distance between the subject chair and light box. Clear, permanent floor markings should indicate 4 meters and 1 meter from the subject chair. Subject chair should be placed at the 0 meter position.

d) Room illumination

The test is performed with the room lights off. With the room lights off, windows covered and light box turned OFF, illumination of the room should be such that not more than 161.4 lux falls on the centre of the chart. Illumination at the centre of the chart should be measured with a lux meter held flat against the centre of the chart. Care should be taken not to tilt the device.

e) Light box illumination

The light box is illuminated with 2 Cool Daylight 20 watt fluorescent tubes. New tubes should be kept on for 96 hours before use, (this does not have to be continuous), and replaced annually. When the light box is turned ON, and the room lights are off, the luminance of the chart should fall between 100 – 150 cd/m^2, corresponding to an illuminance of 320 – 480 lux. Illuminance at the centre and four corners of the chart should be measured with a lux meter. The lux meter should be held facing the chart with the sensor between 10 and 30 cm from the chart surface.

f) Access to previous Refraction results

© 2022 Dunbar HMP et al. *JAMA Ophthalmology.*

During all follow up visits, technicians must have access to the Refraction Score Sheet from the previous study visit.

g) Assessment of health risk

Care is taken to avoid skin cross contamination: the examiner should clean his or her hands with alcohol hand rub before and after contact with each subject. All equipment that comes in contact with the patient should be cleaned with alcohol wipes before and after use.

4.3.2 Preparing the subject

a) Sit the subject in the subject chair in such a way that their eyes are aligned with the 0 metre floor marking. Position the light box so that its front panel is in line with the 4 metre floor marking.

b) Insert ETDRS chart R in the light box. The height of the chart should be such that the top of the third row of letters is 124.5 cm (+/- 5 cm) from the floor. Chart R is used for refracting both eyes.

c) Always test the right eye first. Cover the subject's left eye with a cotton pad and hold in place with surgical tape. Ensure no light can enter the non-study eye. The left eye will be tested immediately after the right eye. When testing the left eye, cover the subject's right eye with a cotton pad and hold in place with surgical tape.

d) Disinfect trial frame with alcohol wipes and allow to dry. Place on subject's head and ensure a comfortable fit. Adjust the distance between and height of the lens holders so that the subject's pupils are centred within the lens holders.

e) Determine starting point lenses for refraction.

At Screening Visit 1: If subject wears distance spectacles, use the prescription of these as a starting point for refraction. Using a focimeter or lensmeter, measure the prescription of the spectacles and place the corresponding trial lenses in the trial frame. Use negative cylinder format. If the subject does not use distance spectacles, perform autorefraction or retinoscopy on both eyes and place the corresponding trial lenses in the trial frame. Use negative cylinder format.

At Baseline Visit 2: No refraction is required. Best Corrected Visual Acuity should be measured using the 4m refraction result found during the Screening Visit 1.

© 2022 Dunbar HMP et al. *JAMA Ophthalmology.*

This can be found on the Refraction Score Sheet completed at the Screening study visit.

At Study Visit 3: Refraction is required. Use the 4m refraction result found at Screening Visit 1 as a starting point. This can be found on the Refraction Score Sheet completed at the Screening study visit.

At Study Visits 4 – 9: Refraction is required. Use the 4m refraction result found at the previous visit as a starting point. This can be found on the Refraction Score Sheet completed at the previous study visit.

At all visits, record the starting point lenses used on the Refraction Score Sheet for the right eye and left eye.

4.3.3 Description of the Procedure

a) Explain the procedure to the subject: "In a moment I will ask you to look at a letter chart whilst I show you some different lenses and ask you some questions about whether these lenses make the letters clearer, more blurred or whether they do not change the appearance of the letters. This will allow me to determine the lenses that give you the best vision to read the chart. I have positioned the chart at the correct distance from you. Do not lean forward during the test. If you lean forward, I will stop testing and ask you to move back to your original position. I will measure your right eye first, followed by your left eye. Do you understand?"

b) The subject should confirm they have understood the above before proceeding. If they have any questions or seek further clarification, respond accordingly. If they do not understand the procedure, repeat step a). Only move onto step c) when the subject confirms they have understood.

c) Ensure the room lights are off and light box is on.

d) Measure the subject's initial visual acuity using the starting lenses appropriate for the visit in the trial frame (see 4.3.2 e).

e) If the subject reads 9 letters or more correctly on ETDRS chart R from 4 metres, perform the refraction at 4 metres. If the subject reads fewer than 9 letters correctly from 4 metres or cannot read any letters correctly from 4 metres, perform the refraction at 1 metre and add a +0.75DS trial lens in front of the eye being tested. If at any point

© 2022 Dunbar HMP et al. *JAMA Ophthalmology.*

throughout the refraction the subject reads more than 6 lines of letters from 1 metre, the chart should be repositioned at 4 meters, +0.75DS removed from the trial lens and refraction procedure restarted.

f) Refraction should be performed according to the procedure described below. Offer plus and minus lenses in intervals appropriate to the subject's acuity as described in the Lens Interval Table (see section 5 below). First check spherical power, then cylinder axis followed by cylinder power and finally recheck spherical power.

g) **Check spherical power:** Refer to the Lens Interval Table to select the appropriate lens interval to use (i.e. if initial acuity 20/40 use ±0.50DS, if initial acuity 20/160 use ±1.00DS). Instruct the subject to look at the smallest letters they can see.

1. Offer plus spherical lens first. Ask subject "Does this lens make the letters clearer, more blurred, or do the letters look the same with this lens?" If the subject indicates the letters are clearer, or look the same, add this plus lens to the trial frame and confirm the subject's visual acuity is at least the same as before the lens was inserted. Repeat this step until the subject indicates the plus lens makes the letters more blurred. If the subject indicates that the letters are more blurred, do not add the lens and move to step 2.
2. Offer negative spherical lens second. Ask subject "Does this lens make the letters clearer, more blurred, or do the letters look the same with this lens?" If the subject indicates the letters are clearer, they must demonstrate they can read at least one more letter correctly with the lens in place. Add this negative lens to the lenses in the trial frame only if the subject can read at least one more letter correctly. If they report the letters to be clearer but cannot read at least one more letter, you must not give this lens. Repeat this step until the subject indicates the negative lens makes the letters more blurred or look the same. If the subject indicates that the letters are more blurred or look the same, do not add this lens and move to step 3.
3. Repeat step 1. After repeating step one, move onto step h)

h) **Check cylinder axis:** Refer to the Lens Interval Table to determine whether it is necessary to check the cylinder axis (i.e. if the subject's acuity is worse than 20/400, no cylinder check is required). If it is necessary to check the cylinder axis, refer to the Lens

© 2022 Dunbar HMP et al. *JAMA Ophthalmology.*

Interval Table to determine which Jackson cross cylinder power lens to use (i.e. if the subject's acuity is 20/50 a ±0.50DC Jackson cross cylinder is required, if the subject's acuity is 20/250 a ±1.00DC cross cylinder is required).

1. If it is necessary to check the cylinder axis, select a round letter (O or C) on a row 1 or 2 lines above the lowest row the subject can read. Instruct the subject to look at this letter and say, "In a moment, I will show you two lenses, lens 1 and lens 2. Both lenses may be blurred, but I would like you to tell me which of these two lenses make the letter (O or C) look rounder. Neither lens may make the letter look perfectly round. If the letter looks equally round with lens 1 and lens 2, you may say the letter (O or C) looks the same with lens 1 and lens 2. The letter (O or C) will likely look rounder without lens 1 or lens 2, but I need you to compare lens 1 and lens 2 only. I may repeat this a few times.
2. Position the appropriate Jackson cross cylinder lens so that the handle is in line with the cylinder axis on the cylindrical trial lens (this is lens 1) and ask the subject "Does the letter (O or C) look rounder with lens 1…". Quickly rotate the lens keeping the handle still (this is lens 2) and continue "..or with lens 2, or do they look the same?" Do not remove the Jackson cross cylinder lens until the subject has responded. If the subject requests to see the lenses again, repeat lens presentation. If the subject has a preference between lens 1 and lens 2, rotate the cylindrical lens in the trial frame 5° towards the position of the negative axis on the Jackson cross cylinder lens (indicated by a red line) when held in the subject's preferred position. Repeat the procedure until the subject cannot notice a difference between the two lenses. If necessary, use smaller degree steps to find the location where the subject cannot notice a difference between the two lenses.

i) Check cylinder power: Refer to the Lens Interval Table to determine whether it is necessary to check the cylinder power (i.e. if the subject's acuity is worse than 20/400, no cylinder check is required.) If it is necessary to check the cylinder power, refer to the Lens Interval Table to determine which Jackson cross cylinder power lens to use (i.e. if the subject's acuity is 20/50 a ±0.25DC Jackson cross cylinder is required and

© 2022 Dunbar HMP et al. *JAMA Ophthalmology.*

±0.25DC interval is used, if the subject's acuity is 20/250 a ±1.00DC Jackson cross cylinder is required and a ±1.00DC interval is used).

1. If it is necessary to check the cylinder power, select a round letter (O or C) on a row 1 or 2 lines above the lowest row the subject can read. Instruct the subject to look at this letter and say, "In a moment, I will show you two lenses, lens 1 and lens 2. Both lenses may be blurred, but I would like you to tell me which of these two lenses make the letter (O or C) look clearer. Neither lens may make the letter look perfectly clear. If the letter looks equally clear with both lens 1 and lens 2, you may say the letter looks the same with lens 1 and lens 2. The letter (O or C) will likely look clearer without lens 1 or lens 2, but I need you to compare lens 1 and lens 2 only. I may repeat this a few times.
2. Position the appropriate Jackson cross cylinder lens so that the negative axis (indicated by a red line) is in line with the cylinder axis of the cylindrical trial lens (this is lens 1) and ask the subject "Does the letter (O or C) look clearer with lens 1…". Quickly rotate the lens keeping the handle still (this is lens 2) and continue "…or with lens 2, or do they look the same?" When the Jackson cross cylinder is in position 2, the plus cylindrical axis of the cross cylinder lens (indicated by a white line) will be in line with the cylinder axis of the cylindrical trial lens. If the subject has a preference between lens 1 and lens 2, adjust the power of the cylindrical lens in the trial frame by the power of the cross cylinder lens (i.e. if using a ±0.25DC cross cylinder lens, adjust by 0.25DC, if using a ±1.00DC, adjust by 1.00DC). If lens 1 is preferred (negative cylinder axis, red line), adjust the lens by the appropriate amount in the negative direction. If lens 2 is preferred (positive cylinder axis, white line), adjust the lens by the appropriate amount in the positive direction. Repeat the procedure until the subject cannot notice a difference between the two lenses. Not all subjects will report no difference between lens 1 and lens 2. A subject may report lens 1 is clearer (power adjusted in the negative direction) and in the next presentation that lens 2 is clearer (power adjusted in the positive direction). Conversely a subject may report lens 2 is clearer (power adjusted in the positive direction) and in the next presentation that lens 1 is clearer

© 2022 Dunbar HMP et al. *JAMA Ophthalmology.*

(power adjusted in the negative direction). In either case, the final cylinder power is the least negative cylindrical lens of the two.

j) **Repeat spherical power check:** If the subject's acuity is less than 20/400, move to step k). If the subject's acuity is better than or equal to 20/400, repeat step g). Refer to the Lens Interval Table (see section 5) to select the appropriate lens interval (i.e. if acuity is 20/40 use ±0.25DS, if acuity is 20/160 use ±0.50DS). Instruct the subject to look at the smallest letters they can see.

k) Record the final spherical, cylinder power and cylinder axis result on the Refraction Score Sheet under 'Right Eye 4m Refraction Result'. In order to reduce the incidence of transcription errors, refraction result should be recorded in the following format + / - _ _ . _ _ / - _ _ . _ _ x _ _ _ (i.e. if the spherical lens use was +1.00DS, this should be recorded as +01.00, if the cylinder axis used was 75, this should be recorded as 075).

l) If the refraction was performed at 1 metre, +0.75DS should be deducted from the final lenses in the trial frame i.e. if final lenses at 1 meter were +00.50 / -02.00 x 090, the final result will be -00.25 / -02.00 x 090; if final lenses at 1 meter were -01.00 / -02.00 x 090, the final result will be -01.75 / -02.00 x 090.

m) Regardless of whether refraction was carried out at 4m or 1m, calculate the lenses required to perform visual function testing at 1m (by adding +0.75DS to the 4m result) and record on the Refraction Score Sheet under 'Right Eye 1m trial lenses'.

n) Repeat the above procedure for the left eye.

o) On completion of the procedure, the examiner must sign and print their name on the Refraction Score Sheet.

p) Proceed to Best Corrected Visual Acuity testing.

5. RELATED MATERIALS

Visual Function Testing Certification Examination

Refraction Score Sheets (not included)

Lens Interval Table (below)

© 2022 Dunbar HMP et al. *JAMA Ophthalmology.*

	Check Spherical Power	Check Cylinder Axis	Check Cylinder Power	Recheck Spherical Power
**VA on Chart R**	**Present spherical lens**	**Present Jackson cross cyl lens**	**Present Jackson cross cyl lens**	**Present spherical lens**
**20/80 or better**	+0.50 DS<br>-0.50 DS	±0.50 DC	±0.25 DC	+0.25 DS<br>-0.25 DS
**20/100 – 20/200**	+1.00 DS<br>-1.00 DS	±0.50 DC	±0.50 DC	+0.50 DS<br>-0.50 DS
**20/250 – 20/400**	+2.00 DS<br>-2.00 DS	±1.00 DC	±1.00 DC	+1.00 DS<br>-1.00 DS
**Less than 20/400**	+2.00 DS<br>-2.00 DS	No cylinder test required	No cylinder test required	No recheck of spherical power required

6. ACKNOWLEDGEMENTS

The MACUSTAR consortium receives funding from the Innovative Medicines Initiative 2 Joint Undertaking under grant agreement No 116076. This Joint Undertaking receives support from the European Union's Horizon 2020 research and innovation programme and EFPIA.

More information about the MACUSTAR project will be available shortly on www.macustar.eu.
More information on IMI is available on www.imi.europa.eu.
Disclaimer: The content of this document reflects the authors' view. Neither IMI nor the European Union or EFPIA are responsible for any use that may be made of the information contained herein.

© 2022 Dunbar HMP et al. *JAMA Ophthalmology.*

Best Corrected Visual Acuity

Standard Operating Procedure

MACUSTAR Study
ECR-AMD-2017-13

Distribution

This SOP is part of the Manual of Study Procedures for the Clinical Study MACUSTAR and its distribution is performed by AIBILI in a controlled manner.

1. PURPOSE

This document describes the procedures to perform Best Corrected Visual Acuity for the Clinical Study *"Development of novel clinical endpoints for interventional clinical trials with a*

© 2022 Dunbar HMP et al. *JAMA Ophthalmology.*

regulatory and patient access intention in patients with intermediate age-related macular degeneration (AMD) – MACUSTAR"(Protocol nº ECR-AMD-2017-13) to ensure that a uniform procedure is followed by all clinical sites (CS) participating in the study, in order to obtain comparable and reliable data, as according to International Conference on Harmonization Good Clinical Practice (ICH-GCP). This procedure will be performed to the study subjects according to the Clinical Study Protocol.

2. POLICY/SCOPE

This SOP will be used when measuring best corrected visual acuity, to ensure all procedures are performed consistently. Adherence to this SOP is necessary to ensure accuracy of best corrected visual acuity measurements, which in turn influence the quality of other visual function measures and subsequent data analysis. Adherence to this SOP will also ensure proper treatment of subjects, and that all data are available for future analysis.

3. STAFF TRAINING AND RESPONSIBILITIES

The Principal Investigator is responsible for ensuring that the appropriate personnel for performing Best Corrected Visual Acuity is identified and trained. All technicians should read this procedure before starting the participation in the Study. Clinical Sites are recommended to have a minimum of 2 technicians in the investigational team for this procedure.

Technicians will be certified as follows:

d) Each technician must read this SOP and the SOPs related to Refraction, Moorfields Acuity Test, Low Luminance Acuity, Contrast Sensitivity and Standardised IReST assessments.

e) Each technician will complete the Visual Function Testing Certification prior to undertaking any subject testing. This multiple choice question exam covers the certification process for Refraction, Best Corrected Visual Acuity, Moorfields Acuity Test, Low Luminance Acuity, Contrast Sensitivity and Standardised IReST assessments.

f) A pass mark of 100% is required to be certified to undertake Best Corrected Visual Acuity, Moorfields Acuity Test, Low Luminance Acuity, Contrast Sensitivity and Standardised IReST assessments. The exam can be repeated until a 100% grade is achieved.

4. PROCEDURE

© 2022 Dunbar HMP et al. *JAMA Ophthalmology.*

4.1 Equipment

This procedure requires use of the following equipment:

Early Treatment of Diabetic Retinopathy Study (ETDRS) fluorescent tube illuminated light box with 5-pronged wheel base
Subject chair with backrest in fixed position
ETDRS letter charts 1 and 2
Best Corrected Visual Acuity Score Sheet RE
Best Corrected Visual Acuity Score Sheet LE
Pen
Lux meter
Trial frame
Open aperture trial lens set
Cotton pads
Indirect Ophthalmoscope
Surgical tape
Alcohol hand rub
Alcohol wipes

4.1.1 Equipment Maintenance

The supplier recommendation for the equipment should be followed for maintenance. Only qualified and trained personnel should do service and repair. All interventions in the equipment should be recorded, kept and made available during monitoring visits or audits if requested.

4.2 Subject Information

Oral information regarding the examination that is being to be performed is given to the subject.

4.3 Technical Procedure

Best corrected visual acuity is defined as the smallest letter size a person can read from an acuity chart. This study uses the Early Treatment Diabetic Retinopathy Study (ETDRS) chart. The ETDRS chart is standardised with equal numbers of letters per row; equal spacing of letters on a row; equal spacing between rows; rows are balanced for difficulty; and the chart provides accurate conversion for Snellen and LogMAR scoring. In order to perform best corrected visual acuity measurements, subjects must first have been refracted according to the MACUSTAR Refraction SOP and the results of this refraction must be readily available.

© 2022 Dunbar HMP et al. *JAMA Ophthalmology.*

4.3.1 Considerations before starting tests

b) Subject identification

All data must be recorded on the Best Corrected Visual Acuity Score Sheet RE and Best Corrected Visual Acuity Score Sheet LE. The subject's study identification code, study eye, date of examination and study visit should be recorded where requested.

b) Pupil size

There are no specific pupil size requirements, but pupils must not be dilated.

c) Testing room dimensions

Best corrected visual acuity is carried out in a room that allows a 4 meter test distance between the subject's chair and light box. Clear, permanent floor markings should indicate 4 meters and 1 meter from the subject chair. Subject chair should be placed at the 0 meter position.

d) Room illumination

The test is performed with the room lights off. With the room lights off, windows covered and light box turned OFF, illumination of the room should be such that not more than 161.4 lux falls on the centre of the chart. Illumination at the centre of the chart should be measured with a lux meter held flat against the centre of the chart. Care should be taken not to tilt the device.

e) Light box illumination

The light box is illuminated with 2 Cool Daylight 20 watt fluorescent tubes. New tubes should be kept on for 96 hours before use, (this does not have to be continuous), and replaced annually. When the light box is turned ON and room lights are off, luminance of the chart should fall between 100 – 150 cd/m^2, corresponding to an illuminance of 320 – 480 lux. Illuminance at the centre and four corners of the chart should be measured with a lux meter. The lux meter should be held facing the chart with the sensor between 10 and 30 cm from the chart surface.

f) Access to previous Best Corrected Visual Acuity results

© 2022 Dunbar HMP et al. *JAMA Ophthalmology.*

At follow up visits, technicians must not have access to any previous best corrected visual acuity scores.

a) Assessment of health risk

Care is taken to avoid skin cross contamination: the examiner should clean his or her hands with alcohol hand rub before and after contact with each subject. All equipment that comes in contact with the patient should be cleaned with alcohol wipes before and after use.

4.3.2 Preparing the subject

a) Sit the subject in the subject chair in such a way that their eyes are aligned with the 0 metre floor marking. Position the light box so that the front of the chart is in line with the 4 metre floor marking. Position the light box at the 4 metre floor marking even if refraction was carried out at 1 meter.

b) Always test the right eye first. Cover the subject's left eye with a cotton pad and hold in place with surgical tape. Ensure no light can enter the left eye. The left eye will be tested immediately after the right eye. When testing the left eye cover the subject's right eye with a cotton pad and hold in place with surgical tape.

c) Disinfect trial frame with alcohol wipes and allow to dry. Place on subject's head and ensure a comfortable fit. Adjust the distance between the lens holders and the height of the lens holders so that the subject's pupils are centred within the lens holders.

d) For all study visits except Baseline Visit 2, place the lenses corresponding to the right 4m refraction result obtained at the same study visit in the trial frame in front of the right eye. During Baseline Visit 2, no refraction is required. Therefore during Baseline Visit 2, place the lenses corresponding to the right 4m refraction result found at Screening Visit 1 in the trial frame in front of the right eye. When testing the left eye, place the lenses corresponding to the left 4m refraction result obtained at the same study visit (except during Baseline Visit 2) in the trial frame in front of the left eye. During Baseline Visit 2, place the lenses corresponding to the left 4m refraction result found at Screening Visit 1 in the trial frame in front of the left eye. Always use negative cylinder format for each eye.

e) Insert ETDRS Chart 1 in the light box. Chart 1 must always be used for the right eye. When testing the left eye, insert ETDRS Chart 2 in the light box. Chart 2 must always be used for the left eye. The height of the chart should be such that the top of the third row of letters is 124.5 cm (+/- 5 cm) from the floor. Ensure the subject is not able to look at either chart prior to testing.

© 2022 Dunbar HMP et al. *JAMA Ophthalmology.*

4.3.3 Description of the Procedure

q) Explain the procedure to the subject: "In a moment I will ask you to read letters on a letter chart 4 metres from you. You should start reading from the first letter on the left side of the top line of the chart. If you cannot read sufficient letters at this distance I will move the chart closer to you, but you must first try to read the chart from 4 metres. You should read the letters slowly and steadily. I recommend you do not read faster than 1 letter per second, but you can read slower if you like. There are only letters on this chart, no numbers. Each line has 5 letters. You must not skip a letter. If you are unsure of a letter you should make your best possible guess. As you read the letters, I will record your responses on my record sheet. You get 1 point for each letter read correctly, but you do not lose a point if you read a letter incorrectly. If you give two responses for a letter, for instance you say 'It's either an N or a H', I will stop you and insist you decide between those two letters before you move onto the next letter. If you make a mistake you can correct your answer as long as you have not attempted the next letter, so try not to rush. Once you have moved onto the next letter, you cannot go back and change your response. When the letters get difficult to see, I will encourage you to keep reading and if necessary, make your best possible guess until you have made some errors. I do not expect you to be able to read every letter. I will let you know when you can stop. You can move your eyes and your head in order to help you see the letters, but you must not lean closer to the chart. If you do, I will stop you and ask you to move back to your original position before you continue reading the letters. It is necessary for us to follow this procedure to be sure we have measured the very smallest letter you can read, even though we know it is difficult for you. If we do not follow this procedure, the measurements we record will not be accurate. I will measure your right eye first, followed by your left eye. Do you understand?"

r) The subject should confirm they have understood the above before proceeding. If they have any questions or seek further clarification, respond accordingly. If they do not understand the procedure, repeat step a). Only move onto step c) when the subject confirms they have understood.

s) Ensure the room lights are off and the light box is turned on.

t) Prepare to complete the Best Corrected Visual Acuity Score Sheet RE. Begin in Section 1 of the chart. As the subject reads the letters, circle each letter read correctly. If the subject reads a letter incorrectly, draw a cross through the letter. If a subject indicates they cannot see a letter, you must ask them to make their 'best possible guess". The subject must respond with a letter. "I don't know" or "I can't see it" are not acceptable responses. Only leave letters blank that were not attempted (i.e. letters after the stopping rule described in f) below).

© 2022 Dunbar HMP et al. *JAMA Ophthalmology.*

u) Instruct the subject to begin reading the chart from the top left letter on the chart and complete the Best Corrected Visual Acuity Score Sheet RE as described above. Do not read any letters to the subject during the test. Do not tell the subject if a letter was read correctly or incorrectly. If the subject loses their place, you may indicate the beginning of the line they are on, but you must move away from the chart once the subject starts to read again. Encourage the subject to attempt letters on the following line as long as more than one letter was read correctly on the previous line.

v) If the subject reads 4 letters or more on the chart from the 4 metre position, encourage them to continue until at least 4 out of 5 letters are read incorrectly on a single line. 1 metre testing is not necessary. Total the number of letters read correctly at the end of each row of section 1 and total this column to give the 'total number of letters read correctly at 4m'. Move to step h) if the subject read 4 letters or more correctly on the chart from the 4 metre position. Move to step g) if the subject read fewer than 4 letters correctly on the chart from the 4 metre position.

w) If the subject reads fewer than 4 letters correctly at the 4 metre test distance, move the letter chart to the 1 meter position. Place a +0.75DS lens over the lenses already in the trial frame and record the resulting lenses where indicated in Section 2 of the Best Corrected Visual Acuity Score Sheet RE. Instruct the subject to read the letters again in the same manner, beginning at the top left letter. Record the subject's responses in the same manner as described in step d) in Section 2 of the Best Corrected Visual Acuity Score Sheet RE. Ensure the subject does not lean forward during testing. Testing should be stopped when the subject has attempted the first 6 rows of the chart, or when the subject reads at least 4 out of 5 letters incorrectly on a single line (whichever occurs first). Total the number of letters read correctly at the end of each row of section 2 and total this column to give the 'total number of letters read correctly at 1m'.

x) Complete section 3 of the Best Corrected Visual Acuity Score Sheet RE to calculate the total letter score. Enter the number of letters read correctly at 4 metres in the first box. If this value is 4 or more, enter 30 in the second box. If the number of letters read correctly at 4 metres is fewer than 4, enter 0 in the second box. Enter the number of letters read correctly at 1 metre (if this was carried out) in the third box. If 1 metre testing was not necessary, enter 0 in the third box.

y) Total the numbers in the first, second and third boxes and enter the value in the fourth box. This is the total letter score. If the subject's total letter score is 0 move to step j). If the subject's total letter score is 1 or more, move to step l).

© 2022 Dunbar HMP et al. *JAMA Ophthalmology.*

z) If the subject fails to read any letters correctly at 1 metre (total letter score 0), assess the subject's ability to detect hand movements. With the rooms lights on, move your hand with fingers outstretched in front of the subject from a distance of 50cm randomly in one of two directions; horizontally (side to side) or vertically (up and down) at a constant speed of one back and forth presentation per second. Your hand should contrast against its background. Ask the subject, 'What direction am I moving my hand, up and down or side to side?' Repeat this 5 times. If the subject responds correctly at least 4 out of 5 times, record their Best Corrected Visual Acuity as 'Hand Movements' by ticking the corresponding box in Section 3 on the Best Corrected Visual Acuity Score Sheet RE and move to step l). If the subject cannot detect 'Hand Movements, move to step k).

aa) If the subject fails to detect hand movements from 50cm, assess the subject's ability to perceive light. Light perception should be measured with an indirect ophthalmoscope in a darkened room. Turn the room lights off. Focus the indirect ophthalmoscope 1 metre from the subject, with the rheostat on maximum voltage. Direct the beam in and out of the eye at least 4 times. Ask the subject, "Please tell me when you see a light." If the examiner is convinced the subject sees the light, record their Best Corrected Visual Acuity as 'Perception of Light' by ticking the corresponding box in Section 3 on the Best Corrected Visual Acuity Score Sheet RE. If the examiner is not convinced the subject sees the light, record their best corrected visual acuity as 'No Perception of Light' by ticking the corresponding box in Section 3 on the Best Corrected Visual Acuity Score Sheet RE.

bb) Repeat process for left eye using the Best Corrected Visual Acuity Score Sheet.

cc) On completion of the procedure, the examiner must sign and print their name on the Best Corrected Visual Acuity Score Sheet RE and Best Corrected Visual Acuity Score Sheet LE.

dd) Proceed to Contrast Sensitivity testing.

5. RELATED MATERIALS

Best Corrected Visual Acuity Score Sheets (not included)

Visual Function Testing Certification Examination

6. ACKNOWLEDGEMENTS

The MACUSTAR consortium receives funding from the Innovative Medicines Initiative 2 Joint Undertaking under grant agreement No 116076. This Joint Undertaking receives support from the European Union's Horizon 2020 research and innovation programme and EFPIA.

© 2022 Dunbar HMP et al. *JAMA Ophthalmology.*

More information about the MACUSTAR project will be available shortly on www.macustar.eu.
More information on IMI is available on www.imi.europa.eu.
Disclaimer: The content of this document reflects the authors' view. Neither IMI nor the European Union or EFPIA are responsible for any use that may be made of the information contained herein.

Low Luminance Visual Acuity

Standard Operating Procedure

MACUSTAR Study

© 2022 Dunbar HMP et al. *JAMA Ophthalmology.*

ECR-AMD-2017-13

Distribution

This SOP is part of the Manual of Study Procedures for the Clinical Study MACUSTAR and its distribution is performed by AIBILI in a controlled manner.

1. PURPOSE

This document describes the procedures to perform Low Luminance Visual Acuity for the Clinical Study *"Development of novel clinical endpoints for interventional clinical trials with a regulatory and patient access intention in patients with intermediate age-related macular degeneration (AMD) – MACUSTAR"*(Protocol nº ECR-AMD-2017-13) to ensure that a uniform

© 2022 Dunbar HMP et al. *JAMA Ophthalmology.*

procedure is followed by all clinical sites (CS) participating in the study, in order to obtain comparable and reliable data, as according to International Conference on Harmonization Good Clinical Practice (ICH-GCP). This procedure will be performed to the study subjects according to the Clinical Study Protocol.

2. POLICY/SCOPE

This SOP will be used when measuring Low Luminance Visual Acuity, to ensure all procedures are performed consistently. Adherence to this SOP is necessary to ensure accuracy of Low Luminance Visual Acuity measurements, which in turn influence the quality of subsequent data analysis. Adherence to this SOP will also ensure proper treatment of subjects, and that all data are available for future analysis.

3. STAFF TRAINING AND RESPONSIBILITIES

The Principal Investigator is responsible for ensuring that the appropriate personnel for performing Low Luminance Visual Acuity is identified and trained. All technicians should read this procedure before starting the participation in the Study. Clinical Sites are recommended to have a minimum of 2 technicians in the investigational team for this procedure. Technicians will be certified as follows:

a) Each technician must read this SOP and the SOPs related to Refraction, Best Corrected Visual Acuity, Moorfields Acuity Test, Contrast Sensitivity and Standardised IReST assessments.
b) Each technician will complete the Visual Function Testing Certification Examination prior to undertaking any subject testing. This multiple choice question exam covers the certification process for Refraction, Best Corrected Visual Acuity, Moorfields Acuity Test, Low Luminance Acuity, Contrast Sensitivity and Standardised IReST assessments.
c) A pass mark of 100% is required to be certified to undertake Best Corrected Visual Acuity, Moorfields Acuity Test, Low Luminance Acuity, Contrast Sensitivity and Standardised IReST assessments. The exam can be repeated until a 100% grade is achieved.

4. PROCEDURE

4.1 Equipment

© 2022 Dunbar HMP et al. *JAMA Ophthalmology.*

This procedure requires use of the following equipment:

Early Treatment of Diabetic Retinopathy Study (ETDRS) fluorescent tube illuminated light box with 5-pronged wheel base
Mesopic light box filter
Subject chair with backrest in fixed position
ETDRS letter charts 1 and 2
Low Luminance Visual Acuity Score Sheet RE
Low Luminance Visual Acuity Score Sheet LE
Pen
Lux meter
Trial frame
Open aperture trial lens set
Indirect ophthalmoscope
Cotton pads
Surgical tape
Alcohol hand rub
Alcohol wipes

4.1.1 Equipment Maintenance

The supplier recommendation for the equipment should be followed for maintenance. Only qualified and trained personnel should do service and repair. All interventions in the equipment should be recorded, kept and made available during monitoring visits or audits if requested.

4.2 Subject Information

Oral information regarding the examination that is being to be performed is given to the subject.

4.3 Technical Procedure

Low Luminance Visual Acuity is defined as the smallest letter size a person can read from an acuity chart under low luminance conditions (3cd/m^2). This study uses the Early Treatment Diabetic Retinopathy Study (ETDRS) chart. The ETDRS chart is standardised with equal numbers of letters per row; equal spacing of letters on a row; equal spacing between rows; rows are balanced for difficulty; and the chart provides accurate conversion for Snellen and LogMAR scoring. In order to perform Low Luminance Visual Acuity measurements, subjects must first have been refracted according to the MACUSTAR Refraction SOP and the results of this refraction must be readily available.

© 2022 Dunbar HMP et al. *JAMA Ophthalmology.*

4.3.1 Considerations before starting tests

a) **Subject identification**

All data must be recorded on the Low Luminance Visual Acuity Score Sheet RE and Low Luminance Visual Acuity Score Sheet LE. The subject's study identification code, study eye, date of examination and study visit should be recorded where requested.

b) Pupil size

There are no specific pupil size requirements, but pupils must not be dilated.

c) Testing room dimensions

Low Luminance Visual Acuity is carried out in a room that allows a 4 meter test distance between the subject's chair and lightbox. Clear, permanent floor markings should indicate 4 meters and 1 meter from the subject chair. Subject chair should be placed at the 0 meter position.

d) Room illumination

The test is performed with the room lights off. With the room lights off, windows covered and lightbox turned OFF, illumination of the room should be such that not more than 161.4 lux falls on the centre of the chart. Illumination at the centre of the chart should be measured with a lux meter held flat against the centre of the chart. Care should be taken not to tilt the device.

e) Lightbox illumination

The lightbox is illuminated with 2 Cool Daylight 20 watt fluorescent tubes. New tubes should be kept on for 96 hours before use, (this does not have to be continuous), and replaced annually. Insert mesopic light box filter into light box. When the lightbox is turned ON and room lights off, illuminance of the chart should be below 10 lux. Illuminance at the centre and four corners of the chart should be measured with a lux meter. The lux meter should be held facing the chart with the sensor between 10 and 30 cm from the chart surface.

f) Access to previous Low Luminance Visual Acuity results

© 2022 Dunbar HMP et al. *JAMA Ophthalmology.*

At follow up visits, technicians must not have access to any previous low luminance visual acuity scores.

g) Assessment of health risk

Care is taken to avoid skin cross contamination: the examiner should clean his or her hands with alcohol hand rub before and after contact with each subject. All equipment that comes in contact with the patient should be cleaned with alcohol wipes before and after use.

4.3.2 Preparing the subject

a) Sit the subject in the subject chair in such a way that their eyes are aligned with the 0 metre floor marking. Position the light box so that the front of the chart is in line with the 4 metre floor marking. Position the light box at the 4 metre floor marking even if refraction was carried out at 1 meter.

b) Subject should sit for at least 10 minutes in darkened test room prior to testing to adapt to light level. Record the time the lights were extinguished and the time the test was started on the Low Luminance Visual Acuity Score Sheet.

c) Always test the right eye first. Cover the subject's left eye with a cotton pad and hold in place with surgical tape. Ensure no light can enter the non-study eye. The left eye will be tested immediately after the right eye. When testing the left eye cover the subject's right eye with a cotton pad and hold in place with surgical tape.

d) Disinfect trial frame with alcohol wipes and allow to dry. Place on subject's head and ensure a comfortable fit. Adjust the distance between the lens holders and the height of the lens holders so that the subject's pupils are centred within the lens holders.

e) Place the lenses corresponding to the right 4m refraction result obtained at the same study visit in the trial frame in front of the right eye. Use negative cylinder format. When testing the left eye, place the lenses corresponding to the left 4m refraction result obtained at the same study visit in the trial frame in front of the left eye.

f) Insert ETDRS Chart 1 in the light box and followed by the mesopic light box filter in front of chart 1. Chart 1 must always be used for the right eye. When testing the left eye, insert ETDRS Chart 2 in the light box followed by the mesopic light box filter. Chart 2 must always be used for the left eye. Make sure to turn the lightbox OFF before removing the filter and / or chart so as not to light adapt the patient. The height of the chart should be such that the top of the third row of letters is 124.5 cm (+/- 5 cm) from the floor. Ensure the subject is not able to look at either chart prior to testing.

© 2022 Dunbar HMP et al. *JAMA Ophthalmology.*

4.3.3 Description of the Procedure

a) Explain the procedure to the subject: "In a moment I will ask you to read letters on a letter chart from 4 metres in very low light. You should start reading from the first letter on the left side of the top line of the chart. If you cannot read sufficient letters at this distance I will move the chart closer to you, but you must first try to read the chart from 4 metres. You should read the letters slowly and steadily. I recommend you do not read faster than 1 letter per second, but you can read slower if you like. There are only letters on this chart, no numbers. Each line has 5 letters. You must not skip a letter. If you are unsure of a letter you should make your best possible guess. As you read the letters, I will record your responses on my record sheet. You get 1 point for each letter read correctly, but you do not lose a point if you read a letter incorrectly. If you give two responses for a letter, for instance you say 'It's either an N or a H', I will stop you and insist you decide between those two letters before you move onto the next letter. If you make a mistake you can correct your answer as long as you have not attempted the next letter, so try not to rush. Once you have moved onto the next letter, you cannot go back and change your response. When the letters get difficult to see, I will encourage you to keep reading and if necessary, make your best possible guess until you have made some errors. I do not expect you to be able to read every letter. I will let you know when you can stop. You can move your eyes and your head in order to help you see the letters, but you must not lean closer to the chart. If you do, I will stop you and ask you to move back to your original position before you continue reading the letters. It is necessary for us to follow this procedure to be sure we have measured the very smallest letters you can read, even though we know it is difficult for you. If we do not follow this procedure, the measurements we record will not be accurate. I will measure your right eye first, followed by your left eye. Do you understand?"

b) The subject should confirm they have understood the above before proceeding. If they have any questions or seek further clarification, respond accordingly. If they do not understand the procedure, repeat step a). Only move onto step c) when the subject confirms they have understood.

c) Ensure the room lights are off and light box is on (with filter inserted). Only begin if the subject has been sitting in dim light for at least 10 minutes.

d) Prepare to complete the Low Luminance Visual Acuity Score Sheet RE. Begin in Section 1 of the chart. As the subject reads the letters, circle each letter read correctly. If the subject

© 2022 Dunbar HMP et al. *JAMA Ophthalmology.*

reads a letter incorrectly, draw a cross through the letter. If a subject indicates they cannot see a letter, you must ask them to make their 'best possible guess". The subject must respond with a letter. "I don't know" or "I can't see it" are not acceptable responses. Only leave letters blank that were not attempted (i.e. letters after the stopping rule described in f) below).

e) Instruct the subject to begin reading the chart from the top left letter on the chart and complete the Low Luminance Visual Acuity Score Sheet RE as described in d). Do not read any letters to the subject during the test. Do not tell the subject if a letter was read correctly or incorrectly. If the subject loses their place, you may indicate the beginning of the line they are on, but you must move away from the chart once the subject starts to read again. Encourage the subject to attempt letters on the following line as long as more than one letter was read correctly on the previous line.

f) If the subject reads 4 letters or more on the chart from the 4 metre position, encourage them to continue until at least 4 out of 5 letters are read incorrectly on a single line. 1 metre testing is not necessary. Total the number of letters read correctly at the end of each row of section 1 and total this column to give the 'total number of letters read correctly at 4m'. Move to step h) if the subject read 4 letters or more correctly on the chart from the 4 metre position. Move to step g) if the subject read fewer than 4 letters correctly on the chart from the 4 metre position.

g) If the subject reads fewer than 4 letters correctly at the 4 metre test distance, move the letter chart to the 1 meter position. Place a +0.75DS lens over the lenses already in the trial frame. Instruct the subject to read the letters again in the same manner, beginning at the top left letter. Record the subject's responses in the same manner as described in step d) in Section 2 of the Low Luminance Visual Acuity Score Sheet RE. Ensure the subject does not lean forward during testing. Testing should be stopped when the subject has attempted the first 6 rows of the chart, or when the subject reads at least 4 out of 5 letters incorrectly on a single line (whichever occurs first). Total the number of letters read correctly at the end of each row of section 2 and total this column to give the 'total number of letters read correctly at 1m'.

h) Complete section 3 of the Low Luminance Visual Acuity Score Sheet RE to calculate the total letter score. Enter the number of letters read correctly at 4 metres in the first box. If this value is 4 or more, enter 30 in the second box. If the number of letters read correctly at 4 metres was fewer than 4, enter 0 in the second box. Enter the number of letters read correctly at 1 metre (if this was carried out) in the third box. If 1 metre testing was not necessary, enter 0 in the third box.

© 2022 Dunbar HMP et al. *JAMA Ophthalmology.*

i) Total the numbers in the first, second and third boxes and enter this value in the fourth box. This is the total letter score. If the subject's total letter score is 0 move to step j). If the subject's total letter score is 1 or more, move to step l).

j) If the subject fails to read any letters correctly at 1 metre (total letter score 0), assess the subject's ability to detect hand movements. With the rooms lights off and lightbox on (with filter inserted), move your hand with fingers outstretched in front of the subject from a distance of 50cm randomly in one of two direction; horizontally (side to side) or vertically (up and down) at a constant speed of one back and forth presentation per second. Your hand should contrast against its background. Ask the subject, 'What direction am I moving my hand, up and down or side to side?' Repeat this 5 times. If the subject responds correctly at least 4 out of 5 times, record their Low Luminance Visual Acuity as 'Hand Movements' by ticking the corresponding box in Section 3 on the Low Luminance Visual Acuity Score Sheet RE and move to step l). If the subject cannot detect 'Hand Movements, move to step k).

k) If the subject fails to detect hand movements from 50cm, assess the subject's ability to perceive light. Light perception should be measured with an indirect ophthalmoscope in a darkened room. Turn the room lights off. Focus the indirect ophthalmoscope 1 metre from the subject, with the rheostat on maximum voltage. Direct the beam in and out of the eye at least 4 times. Ask the subject, "Please tell me when you see a light." If the examiner is convinced the subject sees the light, record their Low Luminance Visual Acuity as 'Perception of Light' by ticking the corresponding box in Section 3 on the Low Luminance Visual Acuity Score Sheet RE. If the examiner is not convinced the subject sees the light, record their Low Luminance Visual Acuity as 'No Perception of Light' by ticking the corresponding box in Section 3 on the Low Luminance Visual Acuity Score Sheet RE.

l) Repeat process for left eye using the Low Luminance Visual Acuity Score Sheet.

m) On completion of the procedure, the examiner must sign and print their name on the Low Luminance Visual Acuity Score Sheet RE and Low Luminance Visual Acuity Score Sheet LE.

n) Disinfect and store all equipment.

5. RELATED MATERIALS

Low Luminance Visual Acuity Score Sheets (not included)

Visual Function Testing Certification Examination

6. ACKNOWLEDGEMENTS

© 2022 Dunbar HMP et al. *JAMA Ophthalmology.*

The MACUSTAR consortium receives funding from the Innovative Medicines Initiative 2 Joint Undertaking under grant agreement No 116076. This Joint Undertaking receives support from the European Union's Horizon 2020 research and innovation programme and EFPIA.

More information about the MACUSTAR project will be available shortly on www.macustar.eu.
More information on IMI is available on www.imi.europa.eu.
Disclaimer: The content of this document reflects the authors' view. Neither IMI nor the European Union or EFPIA are responsible for any use that may be made of the information contained herein.

Moorfields Acuity Test

Standard Operating Procedure

MACUSTAR Study

© 2022 Dunbar HMP et al. *JAMA Ophthalmology.*

ECR-AMD-2017-13

Distribution

This SOP is part of the Manual of Study Procedures for the Clinical Study MACUSTAR and its distribution is performed by AIBILI in a controlled manner.

1. PURPOSE

This document describes the procedures to perform Moorfields Acuity Test for the Clinical

© 2022 Dunbar HMP et al. *JAMA Ophthalmology.*

Study *"Development of novel clinical endpoints for interventional clinical trials with a regulatory and patient access intention in patients with intermediate age-related macular degeneration (AMD) – MACUSTAR"*(Protocol nº ECR-AMD-2017-13) to ensure that a uniform procedure is followed by all clinical sites (CS) participating in the study, in order to obtain comparable and reliable data, as according to International Conference on Harmonization Good Clinical Practice (ICH-GCP). This procedure will be performed to the study subjects according to the Clinical Study Protocol.

2. POLICY/SCOPE

This SOP will be used when measuring Moorfields Acuity Test, to ensure all procedures are performed consistently at all visits and across all sites. Adherence to this SOP is necessary to ensure accuracy of Moorfields Acuity Test measurements, which in turn influence the quality of subsequent data analysis. Adherence to this SOP will also ensure proper treatment of subjects, and that all data are available for future analysis.

3. STAFF TRAINING AND RESPONSIBILITIES

The Principal Investigator is responsible for ensuring that the appropriate personnel for performing Moorfields Acuity Test is identified and trained. All technicians should read this procedure before starting the participation in the Study. Clinical Sites are recommended to have a minimum of 2 technicians in the investigational team for this procedure. Technicians will be certified as follows:

a) Each technician must read this SOP and the SOPs related to Refraction, Best Corrected Visual Acuity, Low Luminance Acuity, Contrast Sensitivity and Standardised IReST assessments.
b) Each technician will complete the Visual Function Testing Certification Examination prior to undertaking any subject testing. This multiple choice question exam covers the certification process for Refraction, Best Corrected Visual Acuity, Moorfields Acuity Test, Low Luminance Acuity, Contrast Sensitivity and Standardised IReST assessments.
c) A pass mark of 100% is required. If the pass mark is required to be certified to undertake Best Corrected Visual Acuity, Moorfields Acuity Test, Low Luminance Acuity, Contrast Sensitivity and Standardised IReST assessments. The exam can be repeated until a 100% grade is achieved.

© 2022 Dunbar HMP et al. *JAMA Ophthalmology.*

4) PROCEDURE

4.1 Equipment

This procedure requires use of the following equipment:

Early Treatment of Diabetic Retinopathy Study (ETDRS) fluorescent tube illuminated light box with 5-pronged wheel base
Subject chair with backrest in fixed position
Moorfields Acuity Test charts 1 and 2
Moorfields Acuity Test Score Sheet RE
Moorfields Acuity Test Score Sheet LE
Pen
Lux meter
Trial frame
Open aperture trial lens set
Indirect Ophthalmoscope
Cotton pads
Surgical tape
Alcohol hand rub
Alcohol wipes

4.1.1 Equipment Maintenance

The supplier recommendation for the equipment should be followed for maintenance. Only qualified and trained personnel should do service and repair. All interventions in the equipment should be recorded, kept and made available during monitoring visits or audits if requested.

4.2 Subject Information

Oral information regarding the examination that is being to be performed is given to the subject.

4.3 Technical Procedure

Moorfields Acuity Test (MAT) is a letter chart constructed of high-pass filtered letter targets comprised of a black core surrounded by a white border presented against a grey background. The mean luminance of the letter is similar to that of the background, therefore detection and recognition thresholds for letters are very similar. Close to threshold, these letters vanish, hence their description of 'vanishing optotypes'. MAT is based upon the Early Treatment Diabetic Retinopathy Study (ETDRS) chart. It is

© 2022 Dunbar HMP et al. *JAMA Ophthalmology.*

standardised with equal numbers of letters per row; equal spacing of letters on a row; equal spacing between rows; rows are balanced for difficulty; and the chart provides accurate conversion for Snellen and LogMAR scoring. In order to perform Moorfields Acuity Test measurements, subjects must first have been refracted according to the MACUSTAR Refraction SOP and the results of this refraction must be readily available.

4.3.1 Considerations before starting tests

a) **Subject identification**

All data must be recorded on the Moorfields Acuity Test Score Sheet RE and Moorfields Acuity Test Score Sheet. The subject's study identification code, study eye, date of examination and study visit should be recorded where requested.

b) Pupil size

There are no specific pupil size requirements, but pupils must not be dilated.

c) Testing room dimensions

Moorfields Acuity Test is carried out in a room that allows a 4 meter test distance between the subject's chair and light box. Clear, permanent floor markings should indicate 4 meters and 1 meter from the subject chair. Subject chair should be placed at the 0 meter position.

d) Room illumination

The test is performed with the room lights on and light box illumination off. Room lighting should be adjusted to ensure even illumination across the chart. Luminance of the white areas of the chart should be 85cd/m^2 (acceptable range 60 – 120 cd/m^2). This corresponds to a luminance of 280lux. (acceptable range 265 – 380 lux). Illumination at the centre and four corners of the chart should be measured with a lux meter. The lux meter should be held flat against the chart. Care should be taken not to tilt the device. Room lights can be altered in order to achieve the required illuminance. If additional light sources are used to satisfy the above criterion, the subject must not see the lamps themselves or any mirror-like reflections from the lamps on the surface of the chart. There should be no glare on the chart.

© 2022 Dunbar HMP et al. *JAMA Ophthalmology.*

e) Access to previous Moorfields Acuity Test results

At follow up visits, technicians must not have access to any previous Moorfields Acuity Test scores.

f) Assessment of health risk

Care is taken to avoid skin cross contamination: the examiner should clean his or her hands with alcohol hand rub before and after contact with each subject. All equipment that comes in contact with the patient should be cleaned with alcohol wipes before and after use.

4.3.2 Preparing the subject

a) Sit the subject in the subject chair in such a way that their eyes are aligned with the 0 metre floor marking. Position the light box so that the front of the chart is in line with the 4 metre floor marking. Position the light box at the 4 metre floor marking even if refraction was carried out at 1 meter.

b) Always test the right eye first. Cover the subject's left eye with a cotton pad and hold in place with surgical tape. Ensure no light can enter the non-study eye. The left eye will be tested immediately after the right eye. When testing the left eye cover the subject's right eye with a cotton pad and hold in place with surgical tape.

c) Disinfect trial frame with alcohol wipes and allow to dry. Place on subject's head and ensure a comfortable fit. Adjust the distance between the lens holders and the height of the lens holders so that the subject's pupils are centred within the lens holders.

d) Place the lenses corresponding to the right 4m refraction result obtained at the same study visit in the trial frame in front of the right eye. Use negative cylinder format. When testing the left eye, place the lenses corresponding to the left 4m refraction result obtained at the same study visit in the trial frame in front of the left eye.

e) Insert Moorfields Acuity Test Chart 1 in the light box. Do not illuminate the light box. Chart 1 must always be used for the right eye. When testing the left eye, insert Moorfields Acuity Test Chart 2 in the light box. Chart 2 must always be used for the left eye. The height of the chart should be such that the top of the third row of letters is 124.5 cm (+/- 5 cm) from the floor. Ensure the subject is not able to look at either chart prior to testing.

4.3.3 Description of the Procedure

a) Explain the procedure to the subject: "In a moment I will ask you to read letters on a letter chart 4 metres from you. You should start reading from the first letter on the left side of the top line of the chart. If you cannot read sufficient letters at

© 2022 Dunbar HMP et al. *JAMA Ophthalmology.*

this distance I will move the chart closer to you, but you must first try to read the chart from 4 metres. You should read the letters slowly and steadily. I recommend you do not read faster than 1 letter per second, but you can read slower if you like. There are only letters on this chart, no numbers. Each line has 5 letters. You must not skip a letter. If you are unsure of a letter you should make your best possible guess. As you read the letters, I will record your responses on my record sheet. You get 1 point for each letter read correctly, but you do not lose a point if you read a letter incorrectly. If you give two responses for a letter, for instance you say 'It's either an N or a H', I will stop you and insist you decide between those two letters before you move onto the next letter. If you make a mistake you can correct your answer as long as you have not attempted the next letter, so try not to rush. Once you have moved onto the next letter, you cannot go back and change your response. When the letters get difficult to see, I will encourage you to keep reading and if necessary, make your best possible guess until you have made some errors. I do not expect you to be able to read every letter. I will let you know when you can stop. You can move your eyes and your head in order to help you see the letters, but you must not lean closer to the chart. If you do, I will stop you and ask you to move back to your original position before you continue reading the letters. It is necessary for us to follow this procedure to be sure we have measured the very smallest letter you can read, even though we know it is difficult for you. If we do not follow this procedure, the measurements we record will not be accurate. I will measure your right eye first, followed by your left eye. Do you understand?"

b) The subject should confirm they have understood the above before proceeding. If they have any questions or seek further clarification, respond accordingly. If they do not understand the procedure, repeat step a). Only move onto step c) when the subject confirms they have understood.

c) Ensure the room light are on and light box is off.

d) Prepare to complete the Moorfields Acuity Test Score Sheet RE. Begin in Section 1 of the chart. As the subject reads the letters, circle each letter read correctly. If the subject reads a letter incorrectly, draw a cross through the letter. If a subject indicates they cannot see a letter, you must ask them to make their 'best possible guess". The subject must respond with a letter. "I don't know" or "I can't see it" are not acceptable responses. Only leave letters blank that were not attempted (i.e. letters after the stopping rule described in f).

© 2022 Dunbar HMP et al. *JAMA Ophthalmology.*

e) Instruct the subject to begin reading the chart from the top left letter on the chart and complete the Moorfields Acuity Test Score Sheet RE as described above. Do not read any letters to the subject during the test. Do not tell the subject if a letter was read correctly or incorrectly. If the subject loses their place, you may indicate the beginning of the line they are on, but you must move away from the chart once the subject starts to read again. Encourage the subject to attempt letters on the following line as long as more than one letter was read correctly on the previous line. The subject may report that the letters have disappeared, but they must continue to make their best possible guess until they have read 4 letters incorrectly on a line.

f) If the subject reads 4 letters or more on the chart from the 4 metre position, encourage them to continue until at least 4 out of 5 letters are read incorrectly on a single line. 1 metre testing is not necessary. Total the number of letters read correctly at the end of each row of section 1 and total this column to give the 'total number of letters read correctly at 4m'. Move to step h) if the subject read 4 letters or more correctly on the chart from the 4 metre position. Move to step g) if the subject read fewer than 4 letters correctly on the chart from the 4 metre position.

g) If the subject reads fewer than 4 letters correctly at the 4 metre test distance, move the letter chart to the 1 meter position. Place a +0.75DS lens over the lenses already in the trial frame and record the resulting lenses where indicated in Section 2 of the Moorfields Acuity Test Score Sheet RE. Instruct the subject to read the letters again in the same manner, beginning at the top left letter. Record the subject's responses in the same manner as described in step d) in Section 2 of the Moorfields Acuity Test Score Sheet RE. Ensure the subject does not lean forward during testing. Testing should be stopped when the subject has attempted the first 6 rows of the chart, or when the subject reads at least 4 out of 5 letters incorrectly on a single line (whichever occurs first). Total the number of letters read correctly at the end of each row of section 2 and total this column to give the 'total number of letters read correctly at 1m'.

h) Complete section 3 of the Moorfields Acuity Test Score Sheet RE to calculate the total letter score. Enter the number of letters read correctly at 4 metres in the first box. If this value is 4 or more, enter 30 in the second box. If the number of letters read correctly at 4 metres was fewer than 4, enter 0 in the second box. Enter the number of letters read correctly at 1 metre (if this was carried out) in the third box. If 1 metre testing was not necessary, enter 0 in the third box.

© 2022 Dunbar HMP et al. *JAMA Ophthalmology.*

i) Total the numbers in the first, second and third boxes and enter the value in the fourth box. This is the total letter score. If the subject's total letter score is 0 move to step j). If the subject's total letter score is 1 or more, move to step l).

j) If the subject fails to read any letters correctly at 1 metre (total letter score 0), assess the subject's ability to detect hand movements. With the rooms lights on, move your hand with fingers outstretched in front of the subject from a distance of 50cm randomly in one of two direction; horizontally (side to side) or vertically (up and down) at a constant speed of one back and forth presentation per second. Your hand should contrast against its background. Ask the subject, 'What direction am I moving my hand, up and down or side to side?' Repeat this 5 times. If the subject responds correctly at least 4 out of 5 times, record their Moorfields Acuity Test score as 'Hand Movements' by ticking the corresponding box in Section 3 on the Moorfields Acuity Test Score Sheet and move to step l). If the subject cannot detect 'Hand Movements, move to step k).

k) If the subject fails to detect hand movements from 50cm, assess the subject's ability to perceive light. Light perception should be measured with an indirect ophthalmoscope in a darkened room. Turn the room lights off. Focus the indirect ophthalmoscope 1 metre from the subject, with the rheostat on maximum voltage. Direct the beam in and out of the eye at least 4 times. Ask the subject, "Please tell me when you see a light.". If the examiner is convinced the subject sees the light, record their Moorfields Acuity Test as 'Perception of Light' by ticking the corresponding box in Section 3 on the Moorfields Acuity Test Score Sheet RE. If the examiner is not convinced the subject sees the light, record their Moorfields Acuity Test as 'No Perception of Light' by ticking the corresponding box in Section 3 on the Moorfields Acuity Test Score Sheet RE.

l) Repeat process for left eye using Moorfields Acuity Test Score Sheet LE.

m) On completion of the procedure, the examiner must sign and print their name on the Moorfields Acuity Test Score Sheet RE and Moorfields Acuity Test Score Sheet LE.

n) Proceed to Standardised International Reading Speed Test.

5. RELATED MATERIALS

Moorfields Acuity Test Score Sheets (not included)

Visual Function Testing Certification Examination

6. ACKNOWLEDGEMENTS

© 2022 Dunbar HMP et al. *JAMA Ophthalmology.*

The MACUSTAR consortium receives funding from the Innovative Medicines Initiative 2 Joint Undertaking under grant agreement No 116076. This Joint Undertaking receives support from the European Union's Horizon 2020 research and innovation programme and EFPIA.

More information about the MACUSTAR project will be available shortly on www.macustar.eu.
More information on IMI is available on www.imi.europa.eu.
Disclaimer: The content of this document reflects the authors' view. Neither IMI nor the European Union or EFPIA are responsible for any use that may be made of the information contained herein.

Pelli-Robson Contrast Sensitivity

Standard Operating Procedure

© 2022 Dunbar HMP et al. *JAMA Ophthalmology.*

MACUSTAR Study
ECR-AMD-2017-13

Distribution

This SOP is part of the Manual of Study Procedures for the Clinical Study MACUSTAR and its distribution is performed by AIBILI in a controlled manner.

1. **PURPOSE**

© 2022 Dunbar HMP et al. *JAMA Ophthalmology.*

This document describes the procedures to perform Contrast Sensitivity for the Clinical Study *"Development of novel clinical endpoints for interventional clinical trials with a regulatory and patient access intention in patients with intermediate age-related macular degeneration (AMD) – MACUSTAR"*(Protocol nº ECR-AMD-2017-13) to ensure that a uniform procedure is followed by all clinical sites (CS) participating in the study, in order to obtain comparable and reliable data, as according to International Conference on Harmonization Good Clinical Practice (ICH-GCP). This procedure will be performed to the study subjects according to the Clinical Study Protocol.

2. POLICY/SCOPE

This SOP will be used when measuring Contrast Sensitivity to ensure the procedure is performed consistently at all visits and across all clinical sites. Adherence to this SOP is necessary to ensure accuracy of contrast sensitivity measurements, which in turn influence the quality of subsequent data analysis. Adherence to this SOP will also ensure proper treatment of subjects, and that all data are available for future analysis.

3. STAFF TRAINING AND RESPONSIBILITIES

The Principal Investigator is responsible for ensuring that the appropriate personnel for performing Contrast Sensitivity are identified and trained. All technicians should read this procedure before starting the participation in the Study. Clinical Sites are recommended to have a minimum of 2 technicians in the investigational team for this procedure. Technicians will be certified as follows:

a) Each technician must read this SOP and the SOPs related to Refraction, Best Corrected Visual Acuity, Moorfields Acuity Test, Low Luminance Acuity, and Standardised IReST assessments.
b) Each technician will complete the Visual Function Testing Certification Examination prior to undertaking any subject testing. This multiple choice question exam covers the certification process for Refraction, Best Corrected Visual Acuity, Moorfields Acuity Test, Low Luminance Acuity, Contrast Sensitivity and Standardised IReST assessments.
c) A pass mark of 100% is required to be certified to undertake Best Corrected Visual Acuity, Moorfields Acuity Test, Low Luminance Acuity, Contrast Sensitivity and

© 2022 Dunbar HMP et al. *JAMA Ophthalmology.*

Standardised IReST assessments. The exam can be repeated until a 100% grade is achieved.

4. PROCEDURE

4.1 Equipment

This procedure requires use of the following equipment:

- Front illuminated Pelli Robson Contrast Sensitivity chart 1 and 2
- Subject chair with backrest in fixed position
- Contrast Sensitivity Score Sheet RE
- Contrast Sensitivity Score Sheet LE
- Pen
- Cotton pad
- Surgical tape
- Trial frame
- Open aperture trial lens set
- Rigid 1m rule
- Alcohol hand rub
- Alcohol wipes

4.1.1 Equipment Maintenance

The supplier recommendations for the equipment should be followed for maintenance. Only qualified and trained personnel should do service and repair. Pelli Robson Contrast Sensitivity charts should be stored away from direct sunlight or UV light sources. The Pelli Robson Contrast Sensitivity chart should be free from blemishes or visible marks. If necessary the Pelli Robson Contrast Sensitivity charts can be wiped gently with a soft cloth using a highly dilute solution of mild soap or detergent in water and rinsed with clean water.

All interventions in the equipment should be recorded, kept and made available during monitoring visits or audits if requested.

4.2 Subject Information

Oral information regarding the examination that is being to be performed is given to the subject.

4.3 Technical Procedure

This SOP describes the method for Contrast Sensitivity testing using a Pelli Robson Contrast Sensitivity chart. The Pelli Robson Contrast Sensitivity chart comprises 16 triplets of letters

arranged in 8 rows of two triplets, each subtending 2.8 deg at the test distance of 1 metre. The three letters within each triplet have constant contrast, whereas the contrast across triplets, reading from left to right, and continuing on successive lines, decreases by a constant factor. In order to perform Contrast Sensitivity measurements, subjects must first have been refracted according to the MACUSTAR Refraction SOP and the results of this refraction must be readily available.

4.3.1 Considerations before starting tests

a) Subject identification and data recording

All data must be recorded on the Contrast Sensitivity Score Sheet RE and Contrast Sensitivity Score Sheet LE. The subject's study identification code, study eye, date of examination and study visit should be recorded where requested.

b) Pupil size

There are no specific pupil size requirements, but pupils must not be dilated.

c) Test room dimensions

Contrast Sensitivity is carried out in a room that allows a 1 meter test distance between the participant and Pelli Robson Contrast Sensitivity chart. Clear, permanent floor markings should indicate 1 meter from the subject chair. Subject chair should be placed at the 0 meter position.

d) Room illumination

Contrast Sensitivity should be illuminated as uniformly as possible, so that the luminance of the white areas of the chart is approximately 85cd/m^2 (acceptable range 60 – 120 cd/m^2) with the room lights on. This corresponds to an illuminance of approximately 280 lux (acceptable range 265 – 380 lux). Illuminance at the centre and four corners of the chart should be measured. The lux meter should be held flat against the chart. Care should be taken not to tilt the device. Room lights can be altered in order to achieve the required illuminance. If additional light sources are used to satisfy the above criterion, the subject must not see the lamps themselves or any mirror-like reflections

© 2022 Dunbar HMP et al. *JAMA Ophthalmology.*

from the lamps on the surface of the chart. There should be no glare on the chart.

e) Access to previous Contrast Sensitivity results

At follow up visits, technicians must not have access to any previous contrast sensitivity scores.

f) Assessment of health risk

Care is taken to avoid skin cross contamination: the examiner should clean his or her hands with alcohol hand rub before and after contact with each participant. All equipment should be cleaned with alcohol wipes before and after use.

4.3.2 Preparing the subject

a) Sit the subject in the subject chair in such a way that their eyes are aligned with the 0 metre floor marking. Instruct subject not to lean forward during the test. If subject leans forward, stop testing and re-position the subject 1 metre from the Pelli Robson Contrast Sensitivity chart before resuming testing.

b) Always test the right eye first. Cover the subject's left eye with a cotton pad and hold in place with surgical tape. Ensure no light can enter the covered eye. The left eye will be tested immediately after the right eye. When testing the left eye, cover the subject's right eye with a cotton pad and hold in place with surgical tape.

c) Disinfect trial frame with disinfecting wipes and allow to dry. Place on subject's head and ensure a comfortable fit. Adjust the distance between the lens holders and the height of the lens holders so that the subject's pupils are centred within the lens holders.

d) Place the lenses corresponding to the right 4m refraction result obtained at the same study visit in front of the right eye. Use negative cylinder format. Add a +0.75DS lens over the 4m refraction result. When testing the left eye, place the lenses corresponding to the left 4m refraction result obtained at the same study visit in front of the left eye. Add a +0.75DS lens over the 4m refraction result.

e) Chart position

© 2022 Dunbar HMP et al. *JAMA Ophthalmology.*

Position the Pelli Robson Contrast Sensitivity chart so that the front of the chart is 1 metre from the subject, measured from the subject's eye. The centre of the chart should be hung so that it is in line with the subject's eye. This will be approximately 1112.5cm ± 12.7cm from the floor for most subjects. Pelli Robson Contrast Sensitivity Chart 1 (first triplet 'HSZ') must always be used for the right eye and Chart 2 (first triplet 'VRS') must always be used for the left eye. Ensure the subject does not see the Pelli Robson Contrast Sensitivity Chart before testing.

4.3.3 Description of the Procedure

a) Explain the procedure to the subject: "This test measures your contrast sensitivity, or how sensitive you are to different grey levels. In a moment I will ask you to read a letter chart from 1 meter away. You should not move closer to the chart. The chart displays letters of equal size, arranged in triplets of equal contrast. There are no numbers on the chart. The first three letters on the top left of the chart are the darkest, the next three are slightly paler, and the next three paler still and so on until the letters are almost invisible. In order for us to make measurements, I will ask you to read the letters aloud until you are no longer able to see them. I recommend you do not read faster than 1 letter per second, but you can read slower if you like. If you are unsure of a letter you should make your best possible guess. As you read the letters, I will record your responses on my record sheet. You get 1 point for each letter read correctly, but you do not lose a point if you read a letter incorrectly. If you give 2 two responses for a letter, for instance you say 'It's either an N or a H', I will stop you and insist you decide between those two letters before you move onto the next letter. If you make a mistake you can correct your answer as long as you have not attempted the next letter, so try not to rush. Once you have moved onto the next letter, you cannot go back and change your response. I will encourage you to keep reading and if necessary, make your best possible guess until you have made some errors. When you first think you are unable to read any more letters, take a moment to move your eye back and forth over the area you expect the letters to be, as sometimes you will see letters you previously thought you could not. I do not expect you to

see all the letters on the chart, but I will ask you to guess the letters even when they seem invisible to you. I will record your responses as you read the chart and will let you know when you can stop attempting the letters. It is necessary for us to follow this procedure to be sure we have measured the palest letters you can see. I will test your right eye first, followed by your left eye. Do you understand?”

b) The subject should confirm they have understood the above before proceeding. If they have any questions or seek further clarification, respond accordingly. If they do not understand the procedure, repeat step a). Only move onto step c) when the subject confirms they have understood.

c) Ensure the room lights are on and light box is off.

d) Prepare to complete the Contrast Sensitivity Score Sheet RE. As the subject reads the letters, circle each letter read correctly. If the subject reads a letter incorrectly, draw a cross through the letter. Only leave letters blank that were not attempted (i.e. letters after the stopping rule as described in g) below.

e) Instruct the subject to beginning reading the letters on the chart, starting with the top left letter, at a speed of about 1 letter per second. Do not read any letters to the subject during the test. Do not tell the subject if a letter was read correctly or incorrectly. If the subject loses their place, you may indicate the beginning of the line they are on, but you must move away from the chart once the subject starts to read again.

f) If the subject indicates they cannot see a letter, you must ask them to make their "best possible guess". Remind them to move their eye back and forth over the area they expect the letters to be. The subject must respond with a letter. "I don't know" or "I can't see it" are not acceptable responses.

g) The subject should continue reading letters until 2 out of 3 letters in a triplet are read incorrectly. The reliability of the test depends upon this.

h) Total the number of letters read correctly at the end of each row and total the ‘letters read’ column to give the ‘Total number of letters read correctly at 1m’.

i) Repeat process for left eye.

j) On completion of the procedure, the examiner must sign and print their name on the Contrast Sensitivity Score Sheet RE and Contrast Sensitivity Score Sheet LE.

k) Proceed to Moorfields Acuity Test.

© 2022 Dunbar HMP et al. *JAMA Ophthalmology.*

5. RELATED MATERIALS

Contrast Sensitivity Score Sheets (not included)

Visual Function Testing Certification Examination

6. ACKNOWLEDGEMENTS

The MACUSTAR consortium receives funding from the Innovative Medicines Initiative 2 Joint Undertaking under grant agreement No 116076. This Joint Undertaking receives support from the European Union's Horizon 2020 research and innovation programme and EFPIA.

More information about the MACUSTAR project will be available shortly on www.macustar.eu.
More information on IMI is available on www.imi.europa.eu.
Disclaimer: The content of this document reflects the authors' view. Neither IMI nor the European Union or EFPIA are responsible for any use that may be made of the information contained herein.

International Reading Speed Test (IReST)

Standard Operating Procedure

© 2022 Dunbar HMP et al. *JAMA Ophthalmology.*

MACUSTAR Study
ECR-AMD-2017-13

Distribution

This SOP is part of the Manual of Study Procedures for the Clinical Study MACUSTAR and its distribution is performed by AIBILI in a controlled manner.

1. **PURPOSE**

© 2022 Dunbar HMP et al. *JAMA Ophthalmology.*

This document describes the procedures to perform a standardised assessment of reading performance using the International Reading Speed Test (IReST) for the Clinical Study *“Development of novel clinical endpoints for interventional clinical trials with a regulatory and patient access intention in patients with intermediate age-related macular degeneration (AMD) – MACUSTAR”*(Protocol nº ECR-AMD-2017-13) to ensure that a uniform procedure is followed by all clinical sites (CS) participating in the study, in order to obtain comparable and reliable data, as according to International Conference on Harmonization Good Clinical Practice (ICH-GCP). This procedure will be performed to the study subjects according to the Clinical Study Protocol.

2. POLICY/SCOPE

This SOP will be used when measuring Standardised IReST reading performance to ensure the procedure is performed consistently at all visits and across all clinical sites. Adherence to this SOP is necessary to ensure accuracy of Standardised IReST assessment, which in turn influences the quality of subsequent data analysis. Adherence to this SOP will also ensure proper treatment of subjects and that all data are available for analysis.

3. STAFF TRAINING AND RESPONSIBILITIES

The Principal Investigator is responsible for ensuring the appropriate personnel for performing Standardised IReST are identified and trained. All technicians should read this procedure before starting the participation in the Study. Clinical Sites are recommended to have a minimum of 2 technicians in the investigational team for this procedure. Technicians will be certified as follows:

a) Each technician must read this SOP and the SOPs related to Refraction, Best Corrected Visual Acuity, Moorfields Acuity Test, Low Luminance Acuity, and Contrast Sensitivity assessments.
b) Each technician will complete the Visual Function Testing Certification Examination prior to undertaking any subject testing. This multiple choice question exam covers the certification process for Refraction, Best Corrected Visual Acuity, Moorfields Acuity Test, Low Luminance Acuity, Contrast Sensitivity and Standardised IReST assessments. Refer to the MACUSTAR Refraction SOP for Visual Function Testing Certification Examination.

© 2022 Dunbar HMP et al. *JAMA Ophthalmology.*

c) A pass mark of 100% is required to be certified to undertake Best Corrected Visual Acuity, Moorfields Acuity Test, Low Luminance Acuity, Contrast Sensitivity and Standardised IReST assessments. The exam can be repeated until a 100% grade is achieved.

4. PROCEDURE

4.1 Equipment

This procedure applies to the following equipment:

10 large print IReST reading cards
10 small print IReST reading cards
Pen
A4 opaque cover sheet
IReST Score Sheets (1 – 10)
Table
Subject chair with backrest in fixed position
Adjustable reading stand
Tape measure
Lux meter
Stopwatch
Trial frame
Open aperture trial lens set
Alcohol hand rub
Alcohol wipes

4.1.1 Equipment Maintenance

The supplier recommendation for the equipment should be followed for maintenance. Only qualified and trained personnel should do service and repair. IReST charts should be stored away from direct sunlight. The IReST chart should be free from blemishes or visible marks. IReST charts can be cleaned with a damp, soft cloth soaked in a very mild solution of detergent and water and rinsed with clean water. All interventions in the equipment should be recorded, kept and made available during monitoring visits or audits if requested.

4.2 Subject Information

Oral information regarding the examination that is being to be performed is given to the subject.

© 2022 Dunbar HMP et al. *JAMA Ophthalmology.*

4.3 Technical Procedure

Standardised IReST reading performance evaluates the subject's reading speed under standardised conditions. The IReST comprises 10 paragraphs of high contrast proportionally spaced texts, each containing about 150 words printed as a single paragraph centred on the page in a column formation to imitate newsprint. The texts have been calibrated to ensure uniform word difficulty and syntactic complexity across 17 languages. They are printed in two sizes (18 pt Times New Roman, referred to here as 'large print' and 9 pt Times New Roman, referred to here as 'small print').

In order to perform Standardised IReST Assessment, subjects must first have been refracted according to the Refraction SOP and the results of this refraction must be readily available.

4.3.1 Considerations before starting tests

a) Subject identification

All data must be recorded on the IReST Score Sheets. The subject's study identification code, study eye, date of examination and study visit should be recorded where requested.

b) Pupil size

There are no specific pupil size requirements, but pupils must not be dilated.

c) Test room dimensions

IReST is carried out in a room with space for a table and chair for the subject, and room for the examiner to stand alongside.

d) Room illumination

IReST is carried out with full room illumination such that the chart illuminance at the level of the IReST chart is between 300 – 400 lux. Additional lighting can be used if room lighting is insufficient. If additional light sources are used to satisfy the above criterion, the subject must not see the lamps themselves or any mirror-like reflections from the lamps on the surface of the chart. There should be no glare on the chart.

Illuminance at the centre of the chart should be measured with a lux meter. Lux meter should be held flat against the centre of the chart. Care should be taken not to tilt the device.

© 2022 Dunbar HMP et al. *JAMA Ophthalmology.*

e) Stopwatch operation

The examiner should ensure they are able to accurately stop and start the stopwatch, and clear the previous reading before taking the next reading.

f) Assessment of health risk

Care is taken to avoid skin cross contamination: the examiner should clean his or her hands with alcohol hand rub before and after contact with each subject. All equipment that comes in contact with the patient should be cleaned with alcohol wipes before and after use.

4.3.2 Preparing the subject

a) Seat the subject at the testing table and ensure they are comfortable.

b) Cover the subject's non-study eye with a cotton pad and hold in place with surgical tape. Ensure no light can enter the non-study eye. Only the study eye will be tested.

c) Disinfect trial frame with alcohol wipes and allow to dry. Place on subject's head and ensure a comfortable fit. Adjust the distance between the lens holders and the height of the lens holders so that the subject's pupil is centred within the lens holders.

d) Place the lenses corresponding to the study eye refraction result obtained at the same study visit in the trial frame in front of the study eye and add a +2.50DS lens. Use negative cylinder format.

e) Place the reading stand on the table in front of the subject, 40cm from the front of the trial frame lenses.

4.3.3 Description of the Procedure

a) Explain the procedure to the subject: "This test measures the speed at which you read text. In a moment I will ask you to read 2 paragraphs of text. Each one is roughly 150 words long. There will be 1 paragraph of large print and 1 paragraph of small print. You will have a break between each paragraph. Each paragraph will be displayed 40 cm from you on a reading stand while you wear the appropriate prescription to focus at that distance. You should not lean closer or further away whilst reading the paragraph. You will read each paragraph with

© 2022 Dunbar HMP et al. *JAMA Ophthalmology.*

your study eye only. I will cover the other eye with a cotton pad. I will place a piece of A4 opaque card in front of the text. There is a black cross on the opaque card. This marks the position of the first word of the paragraph. You should look toward the black cross. When I remove the card, you should read the text aloud, as quickly and as accurately as you can. In order to make sure you are ready to start reading as soon as I remove the card, I will count down from three saying 'Three, Two, One, Start' and will remove the card as I say the word 'Start'. Please begin reading as soon as you hear the word 'Start'. If you have difficulty with a word, skip it and move onto the next word. If you make a mistake, ignore it and move onto the next word. Do not go back to correct it. I will time you with a stopwatch from the moment I remove the opaque card, until you read the final word. If you do not finish the paragraph within 90 seconds, I will ask you to stop reading and the stopwatch will be stopped. I will calculate the speed at which you read the text, taking account of any errors you made. It is important you do not stop reading once you have started. You will have a practise run before the first large print and the first small print text. If you have any questions about this procedure please ask them before we start. If you need any further clarification on the test, please ask me now. Do you understand?"

b) Address any questions the subject has about the procedure and clarify that the subject understands what they must do. If the subject does not understand what is expected of them, repeat step a). Only procedure to step c) when the subject confirms they understand.
c) Ensure the room lights are on.
d) Randomly select 2 large IReST texts and 2 small IReST texts. Texts are numbered 1 to 10. Ensure all 4 texts are different. Assign one large IReST text as the 'Large Practise Text' and the other as the 'Large Test Text'. Assign one small IReST text as a 'Small Practise Text' and the other as the 'Small Test Text'. Do not use any texts used at the previous study visit.
e) Place the 'Large Practise Text' on the reading stand. Cover the text with an opaque A4 sheet making sure the subject does not see the text beneath.
f) Cover the subject's non-study eye with a cotton pad and hold in place with surgical tape. Place the subject's most recent study eye refraction result in the

© 2022 Dunbar HMP et al. *JAMA Ophthalmology.*

trial frame, adding a +2.50DS lens on top. Use negative cylinder format. Place the trial frame on the subject and ensure the subject's pupil is in the centre of the trial lenses by adjusting the vertical and horizontal position of the trial lens holder.

g) With a tape measure, ensure the distance between the IReST text and the front of the trial lenses is 40 cm.

h) Ask the subject to prepare to read the 'Large Practise Text' from this distance and remind them they must not move closer. Remind them to look towards the black cross.

i) Make sure the subject is ready to begin the Practise test. Count down from three saying, 'Three, Two One, Start'. Remove the opaque card as you say the word 'Start' and simultaneously start the stopwatch.

j) When the subject reads the final word, or the stopwatch reads 90 seconds, stop the stopwatch. If the subject performed as required move onto step k). If they did not, repeat the process from step a).

k) Note the text number of 'Large Test Text' and prepare the corresponding IReST Score Sheets (i.e. for IReST Text 1 use IReST Score Sheet 1). Complete all subject identification data as described in 4.3.1 a) on both pages of the score sheet. Circle 'Large' Text Size and the 'Standardised' Test Condition on both pages of the score sheet.

l) Place the 'Large Test Text' on the reading stand and cover it with the opaque card, ensuring the black cross aligns with the start of the text. You must not let the subject see the print before testing. If they do, randomly select a different IReST text and prepare the corresponding IReST Score Sheet as describe in k). Ensure the subject understands that the next trial is the study assessment and not a practise run.

m) On the IReST Score Sheets, record the study eye and enter the lenses used in the trial frame in front of the study eye in negative cylinder format, including the addition of the +2.50DS lens. Tick to confirm a 40 cm working distance was used. If it was not, specify why.

n) Make sure the subject is ready to begin the test and looking at the black cross. Count down from three saying, 'Three, Two, One, Start'. Remove the opaque card as you say the word 'Start' and simultaneously start the stopwatch.

© 2022 Dunbar HMP et al. *JAMA Ophthalmology.*

o) Follow the text on the corresponding IReST Score Card as the subject reads the text. Put a horizontal line through any word or words that are read incorrectly or omitted. If a subject skips (does not read) sections of text, draw double diagonal bars (//) at the end of the last word read correctly and draw double diagonal bars (//) at the end of the last word skipped. Draw double diagonal bars (//) at the end of the last word read within 90 seconds. Draw a horizontal line through all skipped words between the (//) marks.

p) Stop the stopwatch when the final word of the text is read, or when the stopwatch reads 90 seconds (whichever occurs first). When 90 seconds has elapsed, indicate to the subject to stop reading. Draw a horizontal line through all text not read.

q) Record the total time to read the text (in seconds) or 90 seconds if the subject failed to finish, on the IReST Score Sheets under 'Time taken'.

r) Count the number of words read incorrectly or not attempted and record this under 'Number of errors and omissions'. This includes any text skipped and any text not attempted when a subject fails to complete the full text in 90 seconds. Deduct this number from the total number of words in the text, shown on the corresponding IReST Score Sheets. This gives the total number of words read correctly. Before recording this value on the IReST Score Sheets, manually count each word read correctly to ensure this calculation is correct. If there is any discrepancy, repeat this step. Once confident, record this value under 'No. of words read correctly'.

s) Sign and print your name on the IReST Score Sheets where indicated.

t) Confirm the subject is able to continue testing and inform them you will repeat the procedure with a 'Small Practise Text' and 'Small Test Text'.

u) Repeat steps d) to s) using the 'Small Practise Text' and 'Small Test Text' selected in d).

v) Proceed to Low Luminance Visual Acuity.

5. RELATED MATERIALS

IReST Score Sheets (not included)

Visual Function Testing Certification Examination

© 2022 Dunbar HMP et al. *JAMA Ophthalmology.*

6. ACKNOWLEDGEMENTS

The MACUSTAR consortium receives funding from the Innovative Medicines Initiative 2 Joint Undertaking under grant agreement No 116076. This Joint Undertaking receives support from the European Union's Horizon 2020 research and innovation programme and EFPIA.

More information about the MACUSTAR project will be available shortly on www.macustar.eu.
More information on IMI is available on www.imi.europa.eu.
Disclaimer: The content of this document reflects the authors' view. Neither IMI nor the European Union or EFPIA are responsible for any use that may be made of the information contained herein.

© 2022 Dunbar HMP et al. *JAMA Ophthalmology.*

Visual Function Testing Certification Examination

MACUSTAR Study
ECR-AMD-2017-13

Please read and answer the following questions. There are 25 multiple-choice questions. These questions will assess your knowledge and understanding of the MACUSTAR visual function SOPs (Refraction, Best Corrected Visual Acuity, Moorfields Acuity Test, Low Luminance Visual Acuity, Pelli Robson Contrast Sensitivity and International Reading Speed Test (IReST)). The term 'visual acuity testing' refers to all 3 visual acuity tests (Best Corrected Visual Acuity, Moorfields Acuity Test and Low Luminance Visual Acuity). There is only 1 correct answer per question.

A pass mark of 100% is required. The paper can be repeated until a 100% grade is achieved.

© 2022 Dunbar HMP et al. *JAMA Ophthalmology.*

1. A subject is refracted at 1m, what distance should you begin visual acuity testing?

    A. 1m
    B. 1m but move to 4m if subject can read more than 6 lines
    C. 2m
    D. 4m

2. At study visit 4, what 'starting point' lenses should be used for refraction?

    A. Lenses based on autorefraction result
    B. Lenses based on focimetry of existing distance glasses
    C. Lenses found at study visit 3
    D. Lenses found at screening study visit

3. A subject's visual acuity with 'starting point' lenses is 20/100, what lens to do present to them first?

    A. +0.50DS
    B. -0.50DS
    C. -1.00DS
    D. +1.00DS

4. There is a -1.00DC lens in the trial frame and the subject's acuity is 20/40. You are checking the cylindrical power with a ±0.25DC Jackson cross cyl. The subject is looking at a letter 'O' and reports that lens 1 (negative cyl axis) makes the 'O' look clearer. You remove the -1.00DC lens from the trial frame and replace it with:

    A. -1.50DC
    B. -1.50DS
    C. -1.25DS
    D. -1.25DC

5. You are rechecking the spherical power. The subject has 20/200 visual acuity. You present a +1.00DS lens and the subject reports the lens does not make a difference. Do you:

    A. Not add the lens
    B. Present the same lens again
    C. Add the lens
    D. Present a +0.50DS lens

6. You are rechecking the spherical power. The subject has 20/25 visual acuity. You present a -0.25DS lens and the subject reports the lens makes the letters clearer. What is the next thing you do?

© 2022 Dunbar HMP et al. *JAMA Ophthalmology.*

A. Don't add the lens
B. Add the lens
C. Confirm subject can read at least 1 extra letter with an extra -0.25DS before adding the lens
D. Present the lens again

7. You are refracting the patient at 1m. The lenses in the trial frame at the end of the refraction are 00.00 / -00.25 x 125. What is the final refraction result?

A. 00.00 / -00.25 x 125
B. +00.50 / -00.25 x 125
C. -00.75 / -00.25 x 125
D. +00.75 / -00.25 x 125

8. Which of these prescriptions is recorded as per the MACUSTAR Refraction SOP?

A. -02.00 / -1.25 x 020
B. -2.00 / -01.25 x 020
C. -02.00 / -01.25 x 20
D. -02.00 / -01.25 x 020

9. What is the correct order of testing according to MACUSTAR procedures?

A. Best Corrected Visual Acuity, Contrast Sensitivity, Moorfields Acuity Test, International Reading Speed Test, Low Luminance Acuity
B. Low Luminance Acuity, Best Corrected Visual Acuity, Moorfields Acuity Test, Contrast Sensitivity, International Reading Speed Test Moorfields Acuity Test, Best Corrected Visual Acuity, Low Luminance Acuity, Contrast Sensitivity, International Reading Speed Test
C. Best Corrected Visual Acuity, Moorfields Acuity Test, Contrast Sensitivity, Low Luminance Acuity, International Reading Speed Test

10. Which of these visual acuity tests requires the light box to be switched off during testing?

A. Best Corrected Visual Acuity
B. Moorfields Acuity Test
C. Low Luminance Visual Acuity
D. Refraction

11. During Low Luminance Visual Acuity Testing, what is important to remember when switching between letter charts?

A. Chart 2 should be used for the right eye
B. Chart 1 should be used for the left eye
C. Switch on the light box whilst swapping Chart 1 for Chart 2 to avoid light adapting the subject
D. Switch off the light box whilst swapping Chart 1 for Chart 2 to avoid light adapting the subject

12. Which of the following subject instructions related to visual acuity testing is incorrect?

© 2022 Dunbar HMP et al. *JAMA Ophthalmology.*

A. Do not read faster than 1 letter per second.
B. If you make a mistake you can correct your answer as long as you have not attempted the next letter.
C. You can move your eyes and your head in order to help you see the letters, but you must not lean closer to the chart.
D. If you are unsure of a letter you must not guess.

13. When should you instruct the subject to stop reading letters?

A. When they are no longer able to read any more letters
B. When they have read 4 out or 5 letters incorrectly on a single line
C. When they say they have finished
D. When they only read 2 letters correct on a line

14. Which of the following visual acuity score sheets have been filled out correctly; indicate that the SOP was followed correctly?

A.

Chart 1					Letters read
N	C	K	Z	O	5
R	H	S	D	K	5
D	O	V	H	R	5
C	Z	R	H	S	5
O	N	[illegible]	R	C	3
D	K	S	N	V	1
Z	S	O	K	N	0
C	K	D	N	R	
S	R	Z	K	D	
H	Z	O	V	C	

B.

Chart 1					Letters read
N	C	K	Z	O	5
R	H	S	D	K	5
D	O	V	H	R	5
C	Z	R	H	S	5
O	N	H	R	C	4
D	K	S	N	V	2
Z	S	O	K	N	
C	K	D	N	R	
S	R	Z	K	D	
H	Z	O	V	C	
N	V	D	O	K	
V	H	C	N	O	
S	V	H	C	Z	
O	Z	D	V	K	
TOTAL NUMBER OF LETTERS READ CORRECTLY AT 4m					26

© 2022 Dunbar HMP et al. *JAMA Ophthalmology.*

C.

Chart 1					Letters read
N	C	K	Z	O	5
R	H	S	D	K	5
D	O	V	H	R	5
C	Z	R	H	S	5
O	N	H	R	C	3
D	K	S	N	V	1
Z	S	O	K	N	
C	K	D	N	R	
S	R	Z	K	D	
H	Z	O	V	C	
N	V	D	O	K	
V	H	C	N	O	
S	V	H	C	Z	
O	Z	D	V	K	
TOTAL NUMBER OF LETTERS READ CORRECTLY AT 4m					24

D.

Chart 1					Letters read
N	C	K	Z	O	5
R	H	S	D	K	5
D	O	V	H	R	5
C	Z	R	H	S	4
O	N	H	R	C	1
D	K	S	N	V	1
Z	S	O	K	N	
C	K	D	N	R	
S	R	Z	K	D	
H	Z	O	V	C	
N	V	D	O	K	
V	H	C	N	O	
S	V	H	C	Z	
O	Z	D	V	K	
TOTAL NUMBER OF LETTERS READ CORRECTLY AT 4m					21

15. Examine the letter by letter scoring below of a subject who read the letter chart from 4m and then 1m.

4m Test Distance - Chart 1					Letters read
N	C	K	Z	O	2
R	H	S	D	K	0
D	O	V	H	R	
C	Z	R	H	S	
O	N	H	R	C	
D	K	S	N	V	
Z	S	O	K	N	
C	K	D	N	R	
S	R	Z	K	D	
H	Z	O	V	C	
N	V	D	O	K	
V	H	C	N	O	
S	V	H	C	Z	
O	Z	D	V	K	
TOTAL NUMBER OF LETTERS READ CORRECTLY AT 4m					2

1m Test Distance - Chart 1					Letters read
N	C	K	Z	O	5
R	H	S	D	K	5
D	O	V	H	R	5
C	Z	R	H	S	5
O	N	H	R	C	5
D	K	S	N	V	4
TOTAL NUMBER OF LETTERS READ CORRECTLY AT 1m					29

© 2022 Dunbar HMP et al. *JAMA Ophthalmology.*

Which of the final letter score calculations are correct?

A.

TOTAL NUMBER OF LETTERS READ CORRECTLY AT 4m	2
IF MORE THAN 4, ENTER 30 IN BOX TO THE RIGHT, OTHERWISE ADD 0	30
TOTAL NUMBER OF LETTERS READ CORRECTLY AT 1m (if applicable)	29
TOTAL LETTER SCORE (SUM OF THREE ENTRIES ABOVE)	61

B.

TOTAL NUMBER OF LETTERS READ CORRECTLY AT 4m	2
IF MORE THAN 4, ENTER 30 IN BOX TO THE RIGHT, OTHERWISE ADD 0	0
TOTAL NUMBER OF LETTERS READ CORRECTLY AT 1m (if applicable)	29
TOTAL LETTER SCORE (SUM OF THREE ENTRIES ABOVE)	31

C.

TOTAL NUMBER OF LETTERS READ CORRECTLY AT 4m	2
IF MORE THAN 4, ENTER 30 IN BOX TO THE RIGHT, OTHERWISE ADD 0	30
TOTAL NUMBER OF LETTERS READ CORRECTLY AT 1m (if applicable)	0
TOTAL LETTER SCORE (SUM OF THREE ENTRIES ABOVE)	32

D.

TOTAL NUMBER OF LETTERS READ CORRECTLY AT 4m	0
IF MORE THAN 4, ENTER 30 IN BOX TO THE RIGHT, OTHERWISE ADD 0	0
TOTAL NUMBER OF LETTERS READ CORRECTLY AT 1m (if applicable)	29
TOTAL LETTER SCORE (SUM OF THREE ENTRIES ABOVE)	29

16. What is the visual acuity score of this subject?

© 2022 Dunbar HMP et al. *JAMA Ophthalmology.*

4m Test Distance - Chart 1					Letters read
N	C	K	Z	O	
R	H	S	D	K	
D	O	V	H	R	
C	Z	R	H	S	
O	N	H	R	C	
D	K	S	N	V	
Z	S	O	K	N	
C	K	D	N	R	
S	R	Z	K	D	
H	Z	O	V	C	
N	V	D	O	K	
V	H	C	N	O	
S	V	H	C	Z	
O	Z	D	V	K	
**TOTAL NUMBER OF LETTERS READ CORRECTLY AT 4m**					

1m Test Distance - Chart 1					Letters read
N	C	K	Z	O	
R	H	S	D	K	
D	O	V	H	R	
C	Z	R	H	S	
O	N	H	R	C	
D	K	S	N	V	
**TOTAL NUMBER OF LETTERS READ CORRECTLY AT 1m**					

CALCULATING FINAL LETTER SCORE

TOTAL NUMBER OF LETTERS READ CORRECTLY AT 4m	
IF MORE THAN 4, ENTER 30 IN BOX TO THE RIGHT, OTHERWISE ADD 0	
TOTAL NUMBER OF LETTERS READ CORRECTLY AT 1m (if applicable)	
TOTAL LETTER SCORE (SUM OF THREE ENTRIES ABOVE)	

A. 33
B. 24
C. 54
D. 27

17. A subject does not read any letters correctly at 4m or 1m, what should the examiner do next?

A. Assess the subject's ability to perceive light
B. Assess the subject's ability to detect hand movements
C. Give the subject a visual acuity score of 0 and stop testing
D. Give the subject another chance to read the letters

18. You are assessing a subject's ability to detect hand movements. Which of the following statements are true?

A. Extinguish room lights.
B. The subject's visual acuity is hand movements if they respond correctly in at least 2 out of 3 presentations.
C. Move your hand in one of three directions.
D. The subject's visual acuity is hand movements if they respond correctly in at least 4 out of 5 presentations.

19. What is the only permissible viewing distance for contrast sensitivity testing?

© 2022 Dunbar HMP et al. *JAMA Ophthalmology.*

A. 4m
B. 1m
C. At the same distance refraction was carried out
D. 2m

20. What lenses should be used when measuring contrast sensitivity?
A. Refraction result from previous study visit
B. Refraction lenses from same study visit
C. Refraction lenses from same study visit with additional +0.75DS
D. +0.75DS

21. Which of the following contrast sensitivity score sheets have been filled out correctly; indicating that the SOP was followed correctly?

A.

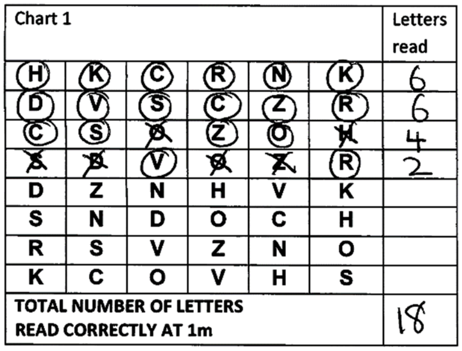

Chart 1						Letters read
H	K	C	R	N	K	6
D	V	S	C	Z	R	6
C	S	O	Z	O	H	4
S	D	V	O	Z	R	2
D	Z	N	H	V	K	
S	N	D	O	C	H	
R	S	V	Z	N	O	
K	C	O	V	H	S	
TOTAL NUMBER OF LETTERS READ CORRECTLY AT 1m						18

B.

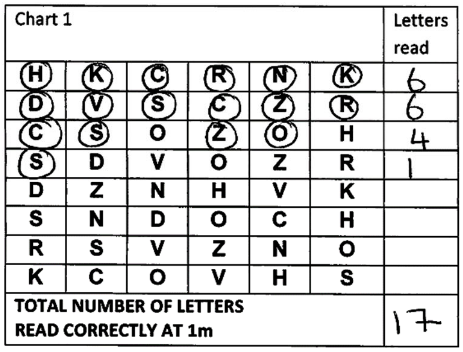

Chart 1						Letters read
H	K	C	R	N	K	6
D	V	S	C	Z	R	6
C	S	O	Z	O	H	4
S	D	V	O	Z	R	1
D	Z	N	H	V	K	
S	N	D	O	C	H	
R	S	V	Z	N	O	
K	C	O	V	H	S	
TOTAL NUMBER OF LETTERS READ CORRECTLY AT 1m						17

C.

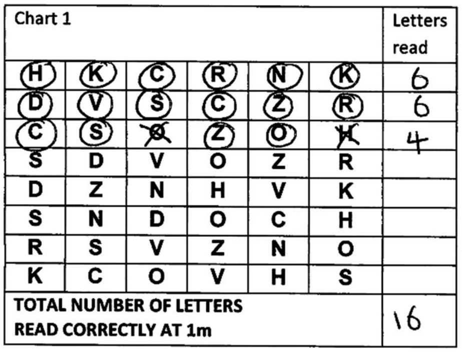

Chart 1						Letters read
H	K	C	R	N	K	6
D	V	S	C	Z	R	6
C	S	O	Z	O	H	4
S	D	V	O	Z	R	
D	Z	N	H	V	K	
S	N	D	O	C	H	
R	S	V	Z	N	O	
K	C	O	V	H	S	
TOTAL NUMBER OF LETTERS READ CORRECTLY AT 1m						16

© 2022 Dunbar HMP et al. *JAMA Ophthalmology.*

D.

Chart 1						Letters read
H	K	C	R	N	K	6
D	V	S	C	Z	R	6
C	S	O	Z	O	H	4
S	D	V	O	Z	R	1
D	Z	N	H	V	K	
S	N	D	O	C	H	
R	S	V	Z	N	O	
K	C	O	V	H	S	
TOTAL NUMBER OF LETTERS READ CORRECTLY AT 1m						17

22. When assessing Habitual IReST, the subject should view the text with:

   A. No spectacles
   B. Refraction lenses from same study visit with an additional +2.50DS lens
   C. Whatever spectacles or visual aid is customary for them when reading at home
   D. Reading spectacles

23. When assessing Standardised IReST, the subject should view the text with:

   A. No spectacles
   B. Refraction lenses from same study visit with an additional +2.50DS lens
   C. Whatever spectacles or visual aid is customary for them when reading at home
   D. Reading spectacles

24. When should the examiner start the stopwatch?

   A. When the A4 opaque card is removed from the IReST text
   B. When the subject reads the first word of the print
   C. When the subject says they are ready to begin
   D. When the examiner is ready

© 2022 Dunbar HMP et al. *JAMA Ophthalmology.*

25. The number of errors or omissions made by this subject was:

A. 3
B. 12
C. 54
D. 50

In a small town a greengrocer had opened a shop that was located above a deep cellar. Every night, mice came in droves out of this cellar into the shop. They ate apples and pears, grapes and nuts and did not spare the vegetables and potatoes either. No goods that were in the shop were safe from the small intrusive rodents between midnight and sunrise. As long as there was noise in the streets at night and cars were driving by, the mice still stayed quietly in the cellar. But as soon as the old clock on the town hall had struck midnight and it became quiet in the street, they came out in droves, enjoyed the sweet fruits and celebrated real feasts, whose remains filled the owner with despair every morning when he entered the shop. So he tried to protect himself against the mice. At first he set up traps all over the shop.

No Words (Total): 156

Clinical Site nº:____

Examiner name: ____

Examiner signature: ____

Date of completion: ____

© 2022 Dunbar HMP et al. *JAMA Ophthalmology.*

CORRECT ANSWERS:

1. D
2. C
3. D
4. D
5. C
6. C
7. C
8. D
9. A
10. B
11. D
12. D
13. B
14. C
15. B
16. D
17. B
18. D
19. B
20. C
21. D
22. C
23. B
24. A
25. C

© 2022 Dunbar HMP et al. *JAMA Ophthalmology.*

ACKNOWLEDGEMENTS

The MACUSTAR consortium receives funding from the Innovative Medicines Initiative 2 Joint Undertaking under grant agreement No 116076. This Joint Undertaking receives support from the European Union's Horizon 2020 research and innovation programme and EFPIA.

More information about the MACUSTAR project will be available shortly on www.macustar.eu.
More information on IMI is available on www.imi.europa.eu.
Disclaimer: The content of this document reflects the authors' view. Neither IMI nor the European Union or EFPIA are responsible for any use that may be made of the information contained herein.

© 2022 Dunbar HMP et al. *JAMA Ophthalmology.*

eTable 1: ICC and Bland Altman (mean deviation and 95% LoA) metrics for each clinical site with 10 or more iAMD participants and the pooled iAMD participants across remaining sites with fewer than 10 iAMD participants. IReST was not performed at site 8 and fewer than 10 iAMD participants completed the IReST at site 2. 0.02 LogMAR = 1 letter. 0.05 LogCS = 1 letter. AMD: age-related macular degeneration; i: intermediate; BCVA: best corrected visual acuity; LLVA: low luminance visual acuity; MAT: Moorfields acuity test; CS: contrast sensitivity; SPS: small print standardised; LPS: large print standardised; LogMAR: logarithm of the minimum angle of resolution; LogCS: logarithm of contrast sensitivity; wpm: words per minute; MD: mean deviation ; CI: confidence interval; LoA: limits of agreement; ICC: Intra-class Correlation Coefficients; IReST: International Reading Speed Test.

Chart-based visual function	Site	ICC (95% CI)	MD (95% CI)	Lower LoA (95% CI)	Upper LoA (95% CI)
BCVA (LogMAR)	1 n = 29	0.99 (0.98, 0.99)	0.01 (-0.02, 0.01)	-0.08 (-0.11, -0.06)	0.07 (0.05, 0.10)
	2 n = 10	0.99 (0.97, 0.99)	0.00 (-0.05, 0.04)	-0.14 (-0.22, -0.05)	0.13 (0.05, 0.21)
	3 n = 11	0.80 (0.43,0.94)	0.01 (-0.05, 0.07)	-0.15 (-0.25, -0.06)	0.18 (0.08, 0.27)
	4 n = 12	0.98 (0.97, 0.99)	-0.01 (-0.06, 0.04)	-0.16 (-0.25, -0.08)	0.14 (0.06, 0.23)
	5 n = 16	0.85 (0.73, 0.92)	0.02 (-0.03, 0.07)	-0.17 (-0.26, -0.08)	0.22 (0.13, 0.31)
	6 n = 20	0.91 (0.80, 0.97)	0.01 (-0.01, 0.02)	-0.05 (-0.08, -0.03)	0.07 (0.04, 0.09)
	7 n = 11	0.90 (0.69, 0.97)	0.01 (-0.04, 0.06)	-0.13 (-0.21, -0.05)	0.15 (0.07, 0.23)
	8 n = 12	0.68 (0.23, 0.90)	0.03 (-0.01, 0.07)	-0.09 (-015, -0.02)	0.15 (0.09, 0.22)
	9 n = 12	0.80 (0.45, 0.94)	0.00 (-0.03, 0.03)	-0.10 (-016, -0.05)	0.10 (0.05, 0.16)
	Remaining n = 34	0.69 (0.47, 0.83)	0.02 (0.00, 0.05)	-0.09 (-0.13, -0.06)	0.14 (0.11, 0.18)
LLVA (LogMAR)	1 n = 29	0.98 (0.96, 0.98)	-0.01 (-0.04, 0.01)	-0.15 (-0.19, -0.10)	0.12 (0.07, 0.16)
	2 n = 10	0.98 (0.96, 0.99)	0.03 (-0.03, 0.08)	-0.12 (-0.22, -0.03)	0.18 (0.08, 0.27)
	3 n = 11	0.78 (0.38, 0.93)	-0.02 (-0.08, 0.04)	-0.18 (-0.28, -0.09)	0.14 (0.48, 0.24)
	4 n = 12	0.97 (0.95, 0.99)	0.04 (-0.02, 0.10)	-0.13 (-0.23, -0.04)	0.21 (0.12, 0.31)
	5 n = 16	0.81 (0.65, 0.90)	0.04 (-0.02, 0.10)	-0.17 (-0.27, -0.07)	0.26 (0.16, 0.35)
	6 n = 20	0.99 (0.98, 0.99)	0.01 (0.00, 0.02)	-0.03 (-0.05, -0.01)	0.05 (0.03, 0.06)
	7 n = 11	0.83 (0.51, 0.95)	0.01 (-0.06, 0.09)	-0.22 (-0.35, -0.08)	0.24 (0.11, 0.38)
	8 n = 12	0.65 (-0.18, 0.88)	0.02 (-0.04, 0.08)	-0.17 (-0.27, -0.06)	0.21 (0.11, 0.31)
	9 n = 12	0.84 (0.56, 0.95)	0.01 (-0.02, 0.05)	-0.09 (-0.15, -0.03)	0.12 (0.06, 0.17)
	Remaining n = 34	0.66 (0.42, 0.81)	0.01 (-0.03, 0.05)	-0.21 (-0.27, -0.14)	0.23 (0.16, -0.30)
MAT (LogMAR)	1 n = 29	0.97 (0.95, 0.81)	0.02 (-0.01, 0.05)	-0.14 (-0.19, -0.08)	0.17 (0.12, 0.22)
	2 n = 10	0.96 (0.91, 0.98)	0.01 (-0.04, 0.06)	-0.13 (-0.22, -0.04)	0.15 (0.06, 0.24)
	3 n = 11	0.87 (0.62, 0.96)	0.01 (-0.03, 0.04)	-0.10 (-0.16, -0.04)	0.11 (0.05, 0.17)
	4 n = 12	0.97 (0.94, 0.98)	0.00 (-0.04, 0.04)	-0.14 (-0.22, -0.06)	0.15 (0.07, 0.23)
	5 n = 16	0.81 (0.66, 0.90)	0.06 (-0.01, 0.12)	-0.19 (-0.30, -0.08)	0.30 (0.19, 0.42)
	6 n = 20	0.97 (0.93, 0.99)	0.02 (0.01, 0.03)	-0.04 (-0.06, -0.01)	0.08 (0.05, 0.10)

© 2022 Dunbar HMP et al. *JAMA Ophthalmology.*

	7<br>n = 11	0.88<br>(0.63, 0.97)	0.02<br>(-0.04, 0.07)	-0.14<br>(-0.23, -0.05)	0.17<br>(0.08, 0.26)
	8<br>n = 12	0.55<br>(0.02, 0.85)	0.04<br>(-0.03, 0.10)	-0.16<br>(-0.27, -0.05)	0.23<br>(0.12, 0.34)
	9<br>n = 12	0.65<br>(0.18, 0.89)	0.02<br>(-0.03, 0.07)	-0.13<br>(-0.21, -0.05)	0.17<br>(0.09, 0.25)
	Remaining<br>n = 34	0.76<br>(0.58, 0.88)	0.02<br>(-0.01, 0.05)	-0.13<br>(-0.17, -0.08)	0.17<br>(0.12, 0.21)
CS<br>(logCS)	1<br>n = 29	0.94<br>(0.90, 0.96)	-0.01<br>(-0.05, -0.03)	-0.20<br>(-0.26, -014)	0.18<br>(0.11, 0.24)
	2<br>n = 10	0.89<br>(0.77, 0.95)	-0.09<br>(-0.20, 0.03)	-0.41<br>(-0.62, -0.21)	0.24<br>(0.04, 0.45)
	3<br>n = 11	0.59<br>(0.04, 0.87)	-0.09<br>(-0.19, 0.02)	-0.38<br>(-0.56, -0.21)	0.21<br>(0.04, 0.39)
	4<br>n = 12	0.89<br>(0.80, 0.94)	-0.01<br>(-0.12, 0.09)	-0.31<br>(-0.48, -0.13)	0.33<br>(0.15, 0.51)
	5<br>n = 16	0.75<br>(0.56, 0.87)	0.03<br>(-0.06, 0.12)	-0.29<br>(-0.44, -0.14)	0.35<br>(0.20, 0.50)
	6<br>n = 20	0.94<br>(0.86, 0.98)	-0.01<br>(-0.03, 0.01)	-0.09<br>(-0.12, -0.05)	0.07<br>(0.04, 0.10)
	7<br>n = 11	0.81<br>(0.46, 0.94)	0.02<br>(-0.04, 0.08)	-0.16<br>(-0.27, -0.06)	0.20<br>(0.09, 0.31)
	8<br>n = 12	0.24<br>(-0.35, 0.70)	0.04<br>(-0.07, 0.15)	-0.37<br>(-0.56, -0.19)	0.30<br>(0.11, 0.48)
	9<br>n = 12	0.51<br>(-0.04, 0.83)	-0.08<br>(-0.16, -0.01)	-0.32<br>(0.46, -0.19)	0.16<br>(0.02, 0.29)
	Remaining<br>n = 34	0.78<br>(0.60, 0.88)	-0.01<br>(-0.05, 0.03)	-0.24<br>(-0.31, -0.17)	0.22<br>(0.15, 0.29)
SPS<br>(wpm)	1<br>n = 29	0.94<br>(0.91, 0.96)	4<br>(-2, 9)	-24<br>(-34, -15)	32<br>(22, 41)
	3<br>n = 11	0.70<br>(0.23, 0.91)	0<br>(-11, 12)	-35<br>(-56, -14)	36<br>(15, 56)
	4<br>n = 12	0.97<br>(0.94, 0.98)	5<br>(-8, 18)	-37<br>(-60, -14)	47<br>(24, 70)
	5<br>n = 16	0.90<br>(0.82, 0.95)	9<br>(1, 17)	-21<br>(-36, -7)	39<br>(25, 53)
	6<br>n = 20	0.92<br>(0.81, 0.97)	-1<br>(-6, 4)	-23<br>(-31, -14)	20<br>(11, 29)
	7<br>n = 10	0.71<br>(0.21, 0.92)	15<br>(6, 24)	-9<br>(-24, 6)	39<br>(24, 54)
	9<br>n = 12	0.78<br>(0.42, 0.93)	2<br>(-7, 11)	-26<br>(-42, -11)	30<br>(14, 45)
	Remaining<br>n = 34	0.69<br>(0.47, 0.83)	1<br>(-8, 10)	-49<br>(-64, -34)	52<br>(36, 67)
LPS<br>(wpm)	1<br>n = 29	0.94<br>(0.91, 0.96)	7<br>(1, 12)	-21<br>(-30, -12)	34<br>(25, 43)
	3<br>n = 11	0.60<br>(0.06, 0.87)	-3<br>(-13, 8)	-33<br>(-52, -15)	28<br>(10, 46)
	4<br>n = 12	0.98<br>(0.96, 0.99)	6<br>(-6, 19)	-32<br>(-54, -11)	45<br>(24, 67)
	5<br>n = 16	0.88<br>(0.78, 0.94)	4<br>(-12, 21)	-57<br>(-85, -29)	65<br>(37, 93)
	6<br>n = 20	0.92<br>(0.81, 0.97)	3<br>(-1, 8)	-14<br>(-21, -6.8)	21<br>(14, 28)
	7<br>n = 10	0.90<br>(0.66, 0.97)	-2<br>(-9, 5)	-22<br>(-35, -10)	18<br>(5.7, 31)
	9<br>n = 12	0.92<br>(0.74, 0.97)	4<br>(-2, 10)	-14<br>(-24, -4)	22<br>(12, 32)
	Remaining<br>n = 32	0.75<br>(0.54, 0.87)	4<br>(-3, 12)	-35<br>(-47, -22)	44<br>(31, 56)

© 2022 Dunbar HMP et al. *JAMA Ophthalmology.*

eTable2: Receiver Operator Characteristic (ROC) analysis summary for no AMD versus early AMD and no AMD versus any AMD. AUC values are provided for each chart-based VF test for initial ROC and cross validated ROCs adjusted for age and sex, with and without the chart-based VF measure included. AUC: Area Under the Curve; CI; confidence interval; CV; cross validated; AMD: age related-macular degeneration; BCVA: best corrected visual acuity; LLVA: low luminance visual acuity; MAT: Moorfields acuity test; CS: contrast sensitivity; SPS: small print standardised; LPS: large print standardised; LogMAR: logarithm of the minimum angle of resolution; LogCS: logarithm of contrast sensitivity; wpm: words per minute.

Chart-based VF	No AMD versus early AMD			No AMD versus any AMD		
	**AUC (95% CI)**	**CV AUC with VF**	**CV AUC without VF**	**AUC (95% CI)**	**CV AUC with VF**	**CV AUC without VF**
BCVA	0.67 (0.55, 0.78)	0.73	0.70	0.74 (0.67, 0.80)	0.78	0.68
LLVA	0.60 (0.47, 0.73)	0.71	0.70	0.75 (0.69, 0.81)	0.78	0.68
LLD	0.52 (0.39, 0.65)	0.70	0.70	0.54 (0.47,0.61)	0.68	0.68
MAT	0.66 (0.54, 0.77)	0.72	0.70	0.75 (0.68, 0.81)	0.77	0.68
CS	0.66 (0.54, 0.77)	0.73	0.70	0.79 (0.73, 0.85)	0.81	0.68
SPS	0.72 (0.61, 0.84)	0.80	0.70	0.66 (0.59, 0.73)	0.73	0.68
LPS	0.71 (0.59, 0.83)	0.79	0.70	0.69 (0.62, 0.76)	0.74	0.67

© 2022 Dunbar HMP et al. *JAMA Ophthalmology.*
