# Supplementary material for: Repeatability and Discriminatory Power of Chart-Based Visual Function Tests in Individuals With Age-Related Macular Degeneration: A MACUSTAR Study Report
Source: JAMA Ophthalmol. 2022 Jun 23;140(8):780–9. doi: 10.1001/jamaophthalmol.2022.2113 (PMC9227684; doi:10.1001/jamaophthalmol.2022.2113)
Supplement: Supplement 2. — The MACUSTAR Consortium [file jamaophthalmol-e222113-s002.pdf]

Supplemental Online Content: Nonauthor Collaborators
*First name, last name, and suffix (if applicable) are required and will appear in PubMed.

*Group Name(s): MACUSTAR CONSORTIUM							
***First Name and Middle Initial(s)**	***Last Name**	***Suffix (eg, Jr, III)**	Academic Degrees	Institution	Location (city, state/province, country)	Role or Contribution, eg, chair, principal investigator	Group (if more than 1 Group listed in the byline) and/or Subgroup (eg, Steering Committee)
Hansjürgen	Agostini						
Francesco	Bandello						
Pier, G.	Basile						
Moritz	Berger						
Camiel, J. F.	Boon						
Michael	Böttger						
Christine	Bouchet						
John, E.	Brazier						
Thomas	Butt						
Claire	Carapezzi						
Jill	Carlton						
Angela	Carneiro						
Arnaud	Charil						
Rita	Coimbra						
José	Cunha-Vaz						
Claudia	Dahlke						
Luis	de Sisternes						
Emily	Fletcher						
Heather	Floyd						
Ruth	Hogg						
Carel	Hoyng						
Jörn	Krätzschmar						
Laura	Kühlewein						
Michael	Larsen						
Anna	Luning						
Cecília, V.	Martinho						
Beatriz, A.	Melício						
Saddek	Mohand-Saïd						
Sandrina	Nunes						

Supplemental Online Content: Nonauthor Collaborators
*First name, last name, and suffix (if applicable) are required and will appear in PubMed.

***First Name and Middle Initial(s)**	***Last Name**	***Suffix (eg, Jr, III)**	Academic Degrees	Institution	Location (city, state/province, country)	Role or Contribution, eg, chair, principal investigator	Group (if more than 1 Group listed in the byline) and/or Subgroup (eg, Steering Committee)
Mariacristina	Parravano						
Daniel	Pauleikhoff						
Maximilian	Pfau						
Susanne, G.	Pondorfer						
Siegfried	Priglinger						
Donna	Rowen						
José A	Sahel						
Daniel	Sanches Fernandes						
Clara I.	Sánchez						
Marlene	Saßmannshausen						
Steffen	Schmitz-Valckenberg						
Hanna	Schrinner-Fenske						
Rufino	Silva						
Adrian	Skelly						
Eric	Souied						
Giovanni	Staurenghi						
Linda	Stöhr						
Diana	Tavares						
Deanna, J.	Taylor						
Sarah	Thiele						
Adnan	Tufail						
Ludmila	Wintergerst						
Christian	Wojek						
